# Research on the stability analysis and safety evaluation method of the surrounding rock of the cross-fault hydraulic tunnel

**DOI:** 10.1038/s41598-025-23163-z

**Published:** 2025-11-12

**Authors:** Ci Kong, Ting Yang, Kuixu Zhu, Xiaomiao Wang, Ming Xiao

**Affiliations:** 1 CISPDR Corporation, Wuhan, 430010 Hubei China; 2 Hubei General Institute of Planning and Design Co., Ltd, Wuhan, 430071 Hubei China; 3 Changjiang Survey, Design and Research Co., Ltd, Planning, Wuhan, 430010 Hubei China; 4 State Key Laboratory of Water Resources Engineering and Management, Wuhan University, Wuhan, 430072 Hubei China

**Keywords:** Cross-fault hydraulic tunnel, Safety evaluation, Constitutive model, Structural surface, Failure modes, Civil engineering, Mechanical engineering

## Abstract

For the cross-fault hydraulic tunnel, rationally simulating the surrounding rock and rock-fault interface, revealing its destabilization risk areas, and evaluating its safety degree are of great significance for its engineering design and construction. Based on the Zienkiewicz-Panda yield criterion and the Drucker-Prager plastic potential function, an elastic-plastic damage constitutive model of the surrounding rock was established, and its rationality was proved. The failure modes of the rock-fault interface were divided into normal disengagement damage, abrasion damage, and snip damage. The peak shear strengths of the structural surface under different shear failure modes were deduced, which were combined with the Desai thin-layer contact element model to form the numerical simulation method of the rock-fault interface. Finally, an evaluation index called the element dangerous coefficient (EDC) was proposed, which is capable of evaluating the element’s safety degree in the whole deformation and damage process. The proposed methods were applied to a cross-fault hydraulic tunnel, and some conclusions were obtained as follows. (1) The value of the EDC gradually decreases from the excavation surface to the interior rock mass, which indicates that the danger degree of the rock mass is gradually decreasing. (2) The rock-fault interface is the weak area, and the contact surface near the excavation surface is disengaged after the excavation, and the EDC value reaches 2.0. (3) The EDC index is capable of quantitatively evaluating the element’s hazard level in the elastic and plastic zones at the same time and giving the element’s hazard coefficient.

## Introduction

Long-distance water transfer projects inevitably suffer from various unfavorable geological structures, such as faults. Compared with the surrounding rock, the fault area is often a high-risk area, which is prone to discontinuous deformation, such as dislocation and slippage of the rock-fault interface, which seriously affects the safety of tunnel construction. Reasonable simulation of the mechanical behavior of the surrounding rock and rock-fault interface, and proposing corresponding safety evaluation methods, are of great significance for analyzing and evaluating the stability of the cross-fault hydraulic tunnel and guiding its engineering design and construction.

 Engineering practices show that the overall instability of tunnels is rare, while local instability caused by structural surfaces occurs frequently, and the mechanical behavior of structural surfaces and their spatial distribution greatly affect the safety and stability of the engineering rock mass. Numerous scholars have conducted in-depth studies on the shear deformation characteristics of joint structural surfaces using joint shear tests^1–6^, but most of them focus on the peak shear strength of the structural surface and do not explicitly give the pre-peak/post-peak constitutive relationship of the structural surface. In terms of the constitutive model of the structural surface, the elastic constitutive model and the elastic-plastic softening model are the two dominant models. Most of the existing elastic constitutive models are only suitable for the pre-peak stage without considering the post-peak softening phenomenon of the structural surface, and only a few scholars^7–9^ give the complete pre-peak and post-peak elastic constitutive relationship of the structural surface. The elastic constitutive model is conceptually simple and easy to implement, but it cannot reflect the plastic deformation of the structural surface. S.C. Desai and K.L. Fishman^10^ developed an elastic-plastic constitutive model of the structural surface. J.G. Wang et al.^11^ developed an elastic-plastic constitutive model of the contact surface based on the limit concept and the associated flow law. L. Xu et al.^12^ established a non-linear elastic-plastic softening model for the rock structural surface by combining a nonlinear elastic model and an elastic-plastic model. M.E. Plesha^4^ developed an incremental elastic-plastic constitutive relationship for the structural surface considering the shear expansion effect and the degradation effect of the undulation angle. H.S. Lee et al.^5^ developed an elastic-plastic constitutive model for the structural surface considering second-order undulation angle degradation effects based on cyclic shear tests in rock joints. J. Deng^13^ established an elastic-plastic damage constitutive model for the structural face based on the principle of effective stress decomposition, taking into account the nonlinear elasticity, plastic hardening, shear expansion and softening as well as the damage characteristics of the structural face. The elastic-plastic damage constitutive model can simulate the structural surface more realistically, but its theory is difficult to implement.

Analyzing and evaluating the safety degree of the hydraulic tunnel is the ultimate aim. To date, there are various qualitative or quantitative destabilization criteria have been developed^14^, they can be categorized as strength criterion, deformation criterion, plastic zone criterion, safety degree criterion, energy criterion, and so on. The strength criterion is based on various rock strength theories, which consider that strength failure occurs when the stress/strain state of the rock mass exceeds a certain limit state, mainly shear damage, tensile damage, and compressive damage^15,16^. However, lots of experiments have proved that the destabilization of the rock mass often occurs in a certain interval of the post-peak softening stage^17^, and the occurrence of strength damage in rock mass does not mean that the rock mass is destabilized, so the strength theory has some difficulties in the application of practical engineering. The deformation criterion refers to the critical displacement of the surrounding rock when instability damage occurs as a criterion. The mainstream method to determine the critical displacement is to conduct a statistical analysis of a large number of engineering disasters based on displacement monitoring data, and then determine the critical displacement^18,19^. However, the critical displacement is related to many factors such as the size of the underground cavern, burial depth, geological conditions, ground stress field, etc., and the displacement values of different parts of a cavern are also different, so it is difficult to determine a critical displacement standard with universal applicability. Plastic zone criterion^20^ refers to an empirical criterion of surrounding rock stability evaluation based on the distribution size and scope of the plastic zone and whether the plastic zone is penetrated or not, which is widely used in engineering, but the penetration of the plastic zone does not mean that the surrounding rock is destabilized, so the plastic zone criterion can only be used as a kind of qualitative standard. The safety degree criterion refers to the definition of the safety coefficient of the rock mass through some mechanical indexes based on the relationship between its present state and critical state, and quantitatively describes its stability through the safety coefficient^21–24^, which has been widely used in slope engineering but not much applied in underground caverns. The energy criterion judges the safety degree of the rock element through the releasable elastic strain energy index and dissipation energy index of the surrounding rock^25^, which can perfectly avoid the complex deformation process of the rock mass. It is a very promising method, but the current research is not mature enough. In addition to the above commonly used criteria, some scholars introduced the mutation theory and established the energy mutation criterion^26^, entropy mutation criterion^27^, displacement mutation criterion^28^, and plastic zone mutation criterion^29^ for the destabilization damage of the surrounding rock. In summary, although numerous surrounding rock instability criteria and stability analysis methods have been formed in the existing studies, a unified consensus has not yet been reached, and a unified standard is lacking.

In view of these, based on the Zienkiewicz-Panda hyperbolic yield criterion (Z-P yield criterion), an elastic-plastic damage constitutive model of the rock mass is established by introducing the Drucker-Prager loading function as the plastic potential function. A nonlinear constitutive relationship of the structural surface considering the pre-peak and post-peak behaviors was established through rigorous theoretical derivation, which was combined with Desai’s thin-layer contact element^30^ to form a numerical analysis method of the structural surface. Based on the yielding approaching index, an evaluation index called the element dangerous coefficient (EDC) was proposed after considering the damage characteristics of the surrounding rock. The proposed model and method were applied to a cross-fault hydraulic tunnel, their rationalities were proved, and some conclusions of interest were drawn.

## Elastic-plastic damage constitutive model of the rock mass

### Decomposition of the effective stress tensor and the elastic-plastic damage constitutive relationship

According to the decomposition principle of the stress tensor, the elastic-plastic stress tensor (hereinafter referred to as the effective stress tensor) in the undamaged state of a rock element can be divided into a volumetric part and a deviatoric part, namely.

1 $${\mathbf{\bar {\sigma }}}_{{ij}}^{{\text{r}}}=\frac{1}{3}{\mathbf{\bar {\sigma }}}_{{kk}}^{{\text{r}}}{\delta _{ij}}+{\mathbf{\bar {s}}}_{{ij}}^{{\text{r}}}$$

where: ${\mathbf{\bar {\sigma }}}_{{ij}}^{{\text{r}}}$ is the effective stress tensor of the rock element, there is ${\text{d}}{\mathbf{\bar {\sigma }}}_{{ij}}^{{\text{r}}}=\varvec{\mathbb{C}}_{{ijkl}}^{{{\text{rep}}}}{\text{d}}{\mathbf{\varepsilon }}_{{kl}}^{{\text{r}}}$; $\varvec{\mathbb{C}}_{{ijkl}}^{{{\text{rep}}}}$ is the elastic-plastic tensor of the rock element; ${\mathbf{\bar {s}}}_{{ij}}^{{\text{r}}}$ is the deviatoric tensor of the effective stress of the rock element; ${\delta _{ij}}$ is the Kronecker symbol.

After yielding of the rock mass, with the accumulation of plastic strain, the stress in the microfracture zone will be released, resulting in the formation of a stress damage zone, and the degree of stress damage can be quantitatively described by the damage coefficient ${d^{\text{r}}}$. To describe this stress damage phenomenon in rock mass, G. Frantziskonis and C.S. Desai^31^ proposed the following elastic-plastic damage constitutive model

2 $${\mathbf{\sigma }}_{{ij}}^{{\text{r}}}=\frac{1}{3}{\mathbf{\bar {\sigma }}}_{{kk}}^{{\text{r}}}{\delta _{ij}}+(1 - {d^{\text{r}}}){\mathbf{\bar {s}}}_{{ij}}^{{\text{r}}}=\frac{{{d^{\text{r}}}}}{3}{\mathbf{\bar {\sigma }}}_{{kk}}^{{\text{r}}}{\delta _{ij}}+(1 - {d^{\text{r}}}){\mathbf{\bar {\sigma }}}_{{ij}}^{{\text{r}}}$$

where:${\mathbf{\sigma }}_{{ij}}^{{\text{r}}}$ is the damage stress tensor of the rock element.

Differentiating Eq. (2) yields the incremental form of the elastic-plastic damage constitutive model of the rock mass, i.e.,

3 $${\text{d}}{\mathbf{\sigma }}_{{ij}}^{{\text{r}}}=(1 - {d^{\text{r}}})\varvec{\mathbb{C}}_{{ijkl}}^{{{\text{rep}}}}{\text{d}}{\mathbf{\varepsilon }}_{{kl}}^{{\text{r}}}+\frac{{{d^{\text{r}}}}}{3}{\delta _{ij}}\varvec{\mathbb{C}}_{{ppkl}}^{{{\text{rep}}}}{\text{d}}{\mathbf{\varepsilon }}_{{kl}}^{{\text{r}}} - {\mathbf{\bar {s}}}_{{ij}}^{{\text{r}}}{\text{d}}({d^{\text{r}}})$$

In the incremental iteration process, making appropriate simplifications and ignoring changes in the damage coefficients, we have

4 $${\text{d}}{\mathbf{\sigma }}_{{ij}}^{{\text{r}}}=(1 - {d^{\text{r}}})\varvec{\mathbb{C}}_{{ijkl}}^{{{\text{rep}}}}{\text{d}}{\mathbf{\varepsilon }}_{{kl}}^{{\text{r}}}+\frac{{{d^{\text{r}}}}}{3}{\delta _{ij}}\varvec{\mathbb{C}}_{{ppkl}}^{{{\text{rep}}}}{\text{d}}{\mathbf{\varepsilon }}_{{kl}}^{{\text{r}}}=\varvec{\mathbb{C}}_{{ijkl}}^{{{\text{rd}}}}{\text{d}}{\mathbf{\varepsilon }}_{{kl}}^{{\text{r}}}$$

where: $\varvec{\mathbb{C}}_{{ijkl}}^{{{\text{rd}}}}$ is the elastic-plastic damage tensor, whose expression is

5 $$\varvec{\mathbb{C}}_{{ijkl}}^{{{\text{rd}}}}=(1 - {d^{\text{r}}})\varvec{\mathbb{C}}_{{ijkl}}^{{{\text{rep}}}}+\frac{{{d^{\text{r}}}}}{3}{\delta _{ij}}\varvec{\mathbb{C}}_{{ppkl}}^{{{\text{rep}}}}$$

### Yield criterion and elastic-plastic tensor

**Fig. 1:**
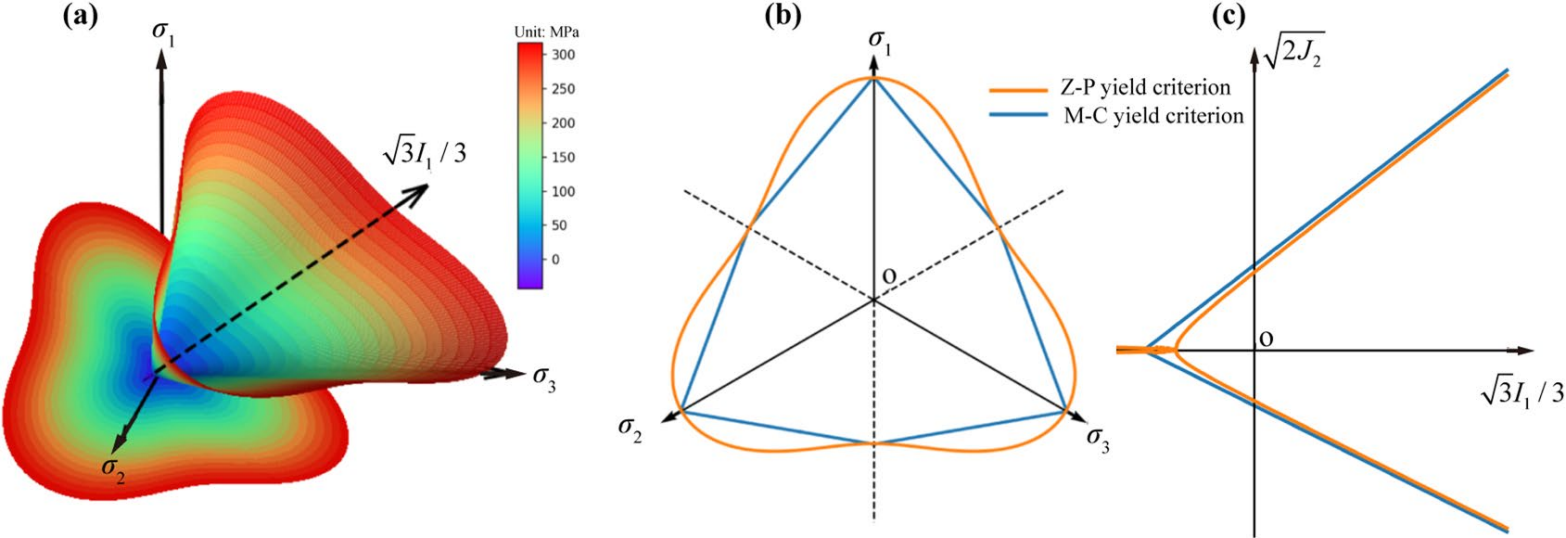
(**a**) Z-P yield surface in the principal stress space and the positional relationship between the Z-P yield surface and the M-C yield surface on the (**b**) π plane and (**c**) the meridian plane.

There exist angular point singularities in the Mohr-Coulomb (M-C) yield surface, which increases the difficulty in numerical processing. Therefore, O.C. Zienkiewicz and C.N. Panda^32^ proposed a hyperbolic Z-P yield criterion by approximating the M-C yield surface by a smooth surface; its expression is

6 $${F^{\text{r}}}{\text{(}}{{\mathbf{\bar {\sigma }}}^{\text{r}}}{\text{) = }}\sqrt { - {\alpha _{\text{r}}}} (\bar {\sigma }_{{\text{m}}}^{{\text{r}}}+\frac{{{\beta _{\text{r}}}}}{{2{\alpha _{\text{r}}}}})+\sqrt {{{({\chi _{\text{r}}})}^2}+{\gamma _{\text{r}}} - \frac{{{\beta _{\text{r}}}^{2}}}{{4{\alpha _{\text{r}}}}}}$$

where: $${\alpha _{\text{r}}}{\text{ = }} - {c_1}{\sin ^2}{\varphi _{\text{r}}}$$; $$\bar {\sigma }_{{\text{m}}}^{{\text{r}}}{\text{ = }}\left( {\bar {\sigma }_{{\text{1}}}^{{\text{r}}}+\bar {\sigma }_{{\text{2}}}^{{\text{r}}}+\bar {\sigma }_{{\text{3}}}^{{\text{r}}}} \right)/3$$; $${\beta _{\text{r}}}{\text{ = }}2{c_1}{c_{\text{r}}}\sin {\varphi _{\text{r}}}\cos {\varphi _{\text{r}}}$$; $${\gamma _{\text{r}}}{\text{ = }}{c_1}\left( {\lambda _{{\text{r}}}^{{\text{2}}}{{\sin }^2}{\varphi _{\text{r}}} - {c_{\text{r}}}^{2}{{\cos }^2}{\varphi _{\text{r}}}} \right)$$; $${\chi _{\text{r}}}{\text{ = }}{{\sqrt {\bar {J}_{{\text{2}}}^{{\text{r}}}} } \mathord{\left/ {\vphantom {{\sqrt {\bar {J}_{{\text{2}}}^{{\text{r}}}} } {g\left( \theta \right)}}} \right. \kern-0pt} {g\left( \theta \right)}}$$; $$g\left( \theta \right){\text{=}}{{2{k_{\text{r}}}} \mathord{\left/ {\vphantom {{2{k_{\text{r}}}} {\left[ {(1+{k_{\text{r}}}) - (1 - {k_{\text{r}}})\sin 3\theta } \right]}}} \right. \kern-0pt} {\left[ {(1+{k_{\text{r}}}) - (1 - {k_{\text{r}}})\sin 3\theta } \right]}}$$; $$\sin 3\theta {\text{ = }}{{ - 3\sqrt 3 \bar {J}_{{\text{3}}}^{{\text{r}}}} \mathord{\left/ {\vphantom {{ - 3\sqrt 3 \bar {J}_{{\text{3}}}^{{\text{r}}}} {2{{\left( {\bar {J}_{{\text{2}}}^{{\text{r}}}} \right)}^{{\raise0.7ex\hbox{$3$} \!\mathord{\left/ {\vphantom {3 2}}\right.\kern-0pt}\!\lower0.7ex\hbox{$2$}}}}}}} \right. \kern-0pt} {2{{\left( {\bar {J}_{{\text{2}}}^{{\text{r}}}} \right)}^{{\raise0.7ex\hbox{$3$} \!\mathord{\left/ {\vphantom {3 2}}\right.\kern-0pt}\!\lower0.7ex\hbox{$2$}}}}}}$$; $${k_{\text{r}}}{\text{ = }}{{\left( {3 - \sin {\varphi _{\text{r}}}} \right)} \mathord{\left/ {\vphantom {{\left( {3 - \sin {\varphi _{\text{r}}}} \right)} {\left( {3+\sin {\varphi _{\text{r}}}} \right)}}} \right. \kern-0pt} {\left( {3+\sin {\varphi _{\text{r}}}} \right)}}$$; $${c_1}{\text{ = }}{{{12} \mathord{\left/ {\vphantom {{12} {\left( {3 - \sin {\varphi _{\text{r}}}} \right)}}} \right. \kern-0pt} {\left( {3 - \sin {\varphi _{\text{r}}}} \right)}}^2}$$. $${c_{\text{r}}}$$ and $${\varphi _{\text{r}}}$$ are the cohesion and the internal friction angle, respectively;$${\lambda _{\text{r}}}$$is a parameter to be determined, along with $${\lambda _{\text{r}}} \to 0$$, Z-P yield surface can be arbitrarily close to M-C yield surface. The positional relationship between the Z-P yield surface and the M-C yield surface is shown in Fig. 1.

In this paper, the non-associated flow rule is adopted and the improved Drucker-Prager loading function is used as the plastic potential function, i.e.

7 $${g^{\text{r}}}\left( {{{{\mathbf{\bar {\sigma }}}}^{\text{r}}}} \right)={\alpha ^{\text{r}}}\bar {I}_{{\text{1}}}^{{\text{r}}}+\sqrt {\bar {J}_{{\text{2}}}^{{\text{r}}}}$$

where: ${\alpha ^{\text{r}}}$ is the expansion parameter. According to the orthogonal flow law, the plastic strain increment of the rock mass is ${\text{d}}{{\mathbf{\varepsilon }}^{{\text{rp}}}}=\Delta {\lambda ^{\text{r}}}\frac{{\partial {g^{\text{r}}}\left( {{{{\mathbf{\bar {\sigma }}}}^{\text{r}}}} \right)}}{{\partial {{{\mathbf{\bar {\sigma }}}}^{\text{r}}}}}$, $\Delta {\lambda ^{\text{r}}}$ is the plastic flow factor. During the deformation process, the following consistency condition needed to be satisfied

8 $$\frac{{\partial {F^{\text{r}}}({{{\mathbf{\bar {\sigma }}}}^{\text{r}}})}}{{\partial {{{\mathbf{\bar {\sigma }}}}^{\text{r}}}}}:{\text{d}}{{\mathbf{\bar {\sigma }}}^{\text{r}}}=\frac{{\partial {F^{\text{r}}}({{{\mathbf{\bar {\sigma }}}}^{\text{r}}})}}{{\partial {{{\mathbf{\bar {\sigma }}}}^{\text{r}}}}}:{\varvec{\mathbb{C}}^{{\text{re}}}}{\text{:}}\left( {{\text{d}}{{\mathbf{\varepsilon }}^{\text{r}}} - \Delta {\lambda ^{\text{r}}}\frac{{\partial {g^{\text{r}}}\left( {{{{\mathbf{\bar {\sigma }}}}^{\text{r}}}} \right)}}{{\partial {{{\mathbf{\bar {\sigma }}}}^{\text{r}}}}}} \right)=0$$

where: ${\varvec{\mathbb{C}}^{{\text{re}}}}$ is the elastic tensor. Thus the plastic flow factor is

9 $$\Delta {\lambda ^{\text{r}}}=\frac{{\frac{{\partial {F^{\text{r}}}}}{{\partial {{{\mathbf{\bar {\sigma }}}}^{\text{r}}}}}:{\varvec{\mathbb{C}}^{{\text{re}}}}{\text{:d}}{{\mathbf{\varepsilon }}^{\text{r}}}}}{{\frac{{\partial {F^{\text{r}}}}}{{\partial {{{\mathbf{\bar {\sigma }}}}^{\text{r}}}}}:{\varvec{\mathbb{C}}^{{\text{re}}}}{\text{:}}\frac{{\partial {g^{\text{r}}}}}{{\partial {{{\mathbf{\bar {\sigma }}}}^{\text{r}}}}}}}$$

Then the elastic-plastic tensor of the rock element can be obtained as follows.

10 $${\varvec{\mathbb{C}}^{{\text{rep}}}}={\varvec{\mathbb{C}}^{{\text{re}}}} - \frac{{\left( {{\varvec{\mathbb{C}}^{{\text{re}}}}:\frac{{\partial {g^{\text{r}}}}}{{\partial {{{\mathbf{\bar {\sigma }}}}^{\text{r}}}}}} \right) \otimes \left( {{\varvec{\mathbb{C}}^{{\text{re}}}}:\frac{{\partial {F^{\text{r}}}}}{{\partial {{{\mathbf{\bar {\sigma }}}}^{\text{r}}}}}} \right)}}{{\frac{{\partial {F^{\text{r}}}}}{{\partial {{{\mathbf{\bar {\sigma }}}}^{\text{r}}}}}:{\varvec{\mathbb{C}}^{{\text{re}}}}{\text{:}}\frac{{\partial {g^{\text{r}}}}}{{\partial {{{\mathbf{\bar {\sigma }}}}^{\text{r}}}}}}}$$

The elastic-plastic damage tensor of the rock mass can be obtained by substituting Eq. (10) into Eq. (5).

### Damage criterion and damage evolution equation

G. Frantziskonis and C.S. Desai^31^ considered the damage coefficient of the rock mass as a monotonic function of the strain tensor, independent of the stress tensor, and considering that elastic deformation does not cause changes in the damage of the structure, it is considered as a monotonic function of the cumulative plastic deviatoric strain, namely

11 $${d^{\text{r}}}={d^{{\text{ru}}}} - {d^{{\text{ru}}}}\exp \left( { - {B_{\text{r}}}{{({\xi _r})}^{{C_{\text{r}}}}}} \right),{\text{ }}{\xi _{\text{r}}}=\int {\sqrt {{\text{d}}{{\mathbf{e}}^{{\text{rp}}}}:{\text{d}}{{\mathbf{e}}^{{\text{rp}}}}} }$$

where: ${d^{{\text{ru}}}}$ is the ultimate damage coefficient of the rock mass; ${B_{\text{r}}}$ and ${C_{\text{r}}}$ are the damage constants; ${{\mathbf{e}}^{{\text{rp}}}}$ is the deviatoric tensor of the plastic strain of the rock mass. ${d^{{\text{ru}}}}$, ${B_{\text{r}}}$ and ${C_{\text{r}}}$ can be determined according to experimental data.

### Verification of the elastic-plastic damage constitutive model

#### Case 1

**Fig. 2:**
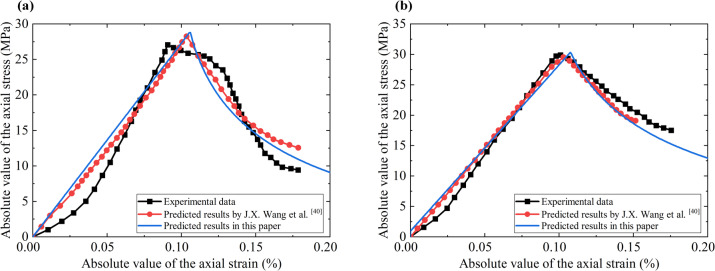
Comparisons between the prediction results by the proposed model with the experimental data, and the prediction results obtained by J.X. Wang et al.^33^ (a) without peripheral pressure and (b) with 2 MPa peripheral pressure.

J.X. Wang et al.^33^ made a cylindrical specimen (50 mm in diameter and 100 mm in height) using rock material collected in the field, and conducted a rock compression test using a three-dimensional rheological experimental system, and the stress-strain curves of the rock without and with peripheral pressure are shown in the black lines in Figs. 2(a) and 2(b), respectively. In addition, J.Z. Wang et al.^33^ also established an elastic-plastic damage constitutive model based on the Drucker-Prager yield criterion to conduct numerical prediction for the rock specimen, and the predicted stress-strain curves are shown as red lines in Figs. 2(a) and 2(b). The proposed model in this paper was also used to conduct numerical prediction for this rock specimen, the prediction results are plotted as blue lines in Figs. 2(a) and 2(b). The calculation parameters are Young’s modulus 27.30 GPa, Poisson’s ratio 0.24, cohesion 12.00 MPa, internal friction angle 32.57°, expansion parameter ${\alpha ^{\text{r}}}$= 0.3, ultimate damage coefficient ${d^{{\text{ru}}}}$ = 0.95, and damage constants ${B_{\text{r}}}$= 300, ${C_{\text{r}}}$=1.0.

From Fig. 2(a), the peak stress predicted by the proposed model in this paper is 28.79 MPa without peripheral pressure, which is very close to the peak stress of 27.10 MPa obtained from the test data. From the stress-strain curve, since the model assumes that the rock mass satisfies Hooke’s law of linear elasticity before yielding, the stress-strain in the pre-peak stage exhibits a linear relationship, which is a certain difference from the test data but perfectly fits the prediction results of the model proposed by J.X. Wang et al.^33^ In the post-peak stage, due to the accumulation of plastic strain, the rock mass damage occurs, and the stress decreases rapidly. The model-predicted post-peak stress-strain curve is basically consistent with the experimental data and the model prediction results obtained by J.X. Wang et al.^33^ From Fig. 2(b), it can be seen that the peak stress of the rock specimen increases after applying a peripheral pressure of 2 MPa, and the peak stress predicted by the model is 30.33 MPa, which is very close to the peak stress of 30.04 MPa obtained from the test data, and the model-predicted stress-strain curves of the pre-peak/post-peak stage match well with the test data and the model prediction of J.X. Wang et al.^33^.

**Fig. 3:**
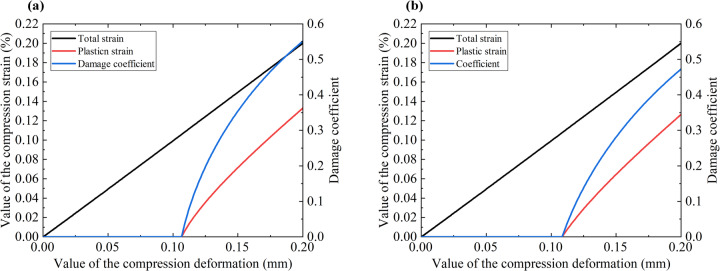
The evolution laws of the predicted total strain, plastic strain, and damage coefficient by the proposed model. (**a**) without peripheral pressure and (**b**) with 2 MPa peripheral pressure.

The evolution laws of the total strain, plastic strain, and damage coefficient of the rock mass predicted by the proposed model in this paper are shown in Fig. 3. It can be seen that when the compression deformation reaches about 0.10 mm, the rock element yields and produces plastic deformation, and then the plastic deformation increases rapidly. With the gradual accumulation of plastic deformation, the rock element undergoes damage, and the damage coefficient increases rapidly, leading to the softening of the material and the reduction of strength. The evolution laws of the plastic strain and damage coefficient without or with peripheral pressure are the same, but after applying peripheral pressure, the damage coefficient decreases significantly, which is also the reason for the increase in rock mass strength. The above results adequately verify the rationality of the elastic-plastic damage model proposed in this paper.

#### Case 2

**Fig. 4:**
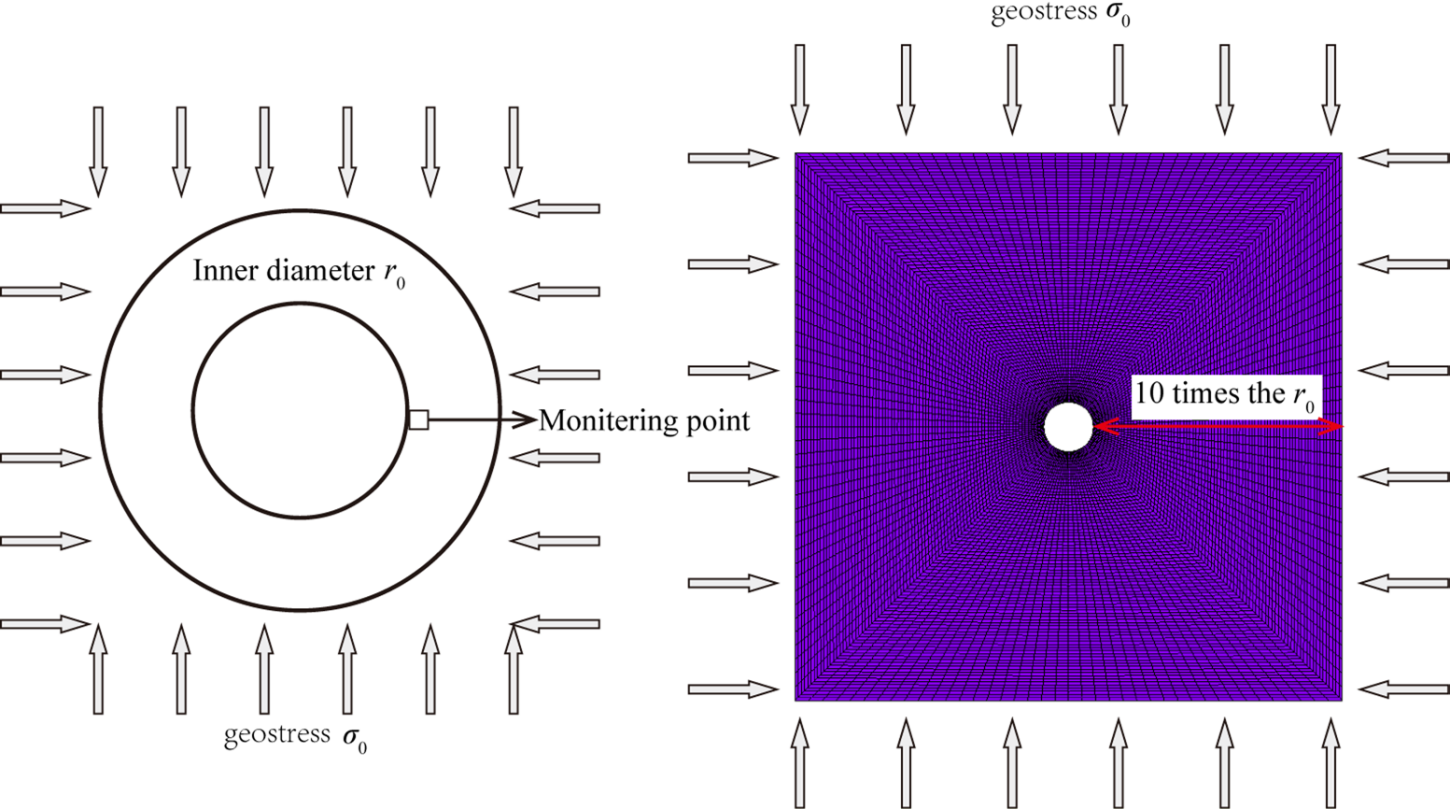
Circular tunnel and its finite element model.

To further validate the model’s validity, numerical predictions were conducted for a circular tunnel depicted in Fig. 4. The predicted plastic zone boundaries and stress distribution patterns from the model were compared with results from an ideal elastoplastic model based on the Mohr-Coulomb criterion. The tunnel has a radius of 3 m, with surrounding rock Young’s modulus of 10 GPa, Poisson’s ratio of 0.25, cohesion of 1.0 MPa, internal friction angle of 30°, and in-situ stress *σ*_₀_ = 20 MPa, expansion parameter ${\alpha ^{\text{r}}}$= 0.3, ultimate damage coefficient ${d^{{\text{ru}}}}$ = 0.95, and damage constants ${B_{\text{r}}}$= 300, ${C_{\text{r}}}$=1.0.

**Fig. 5:**
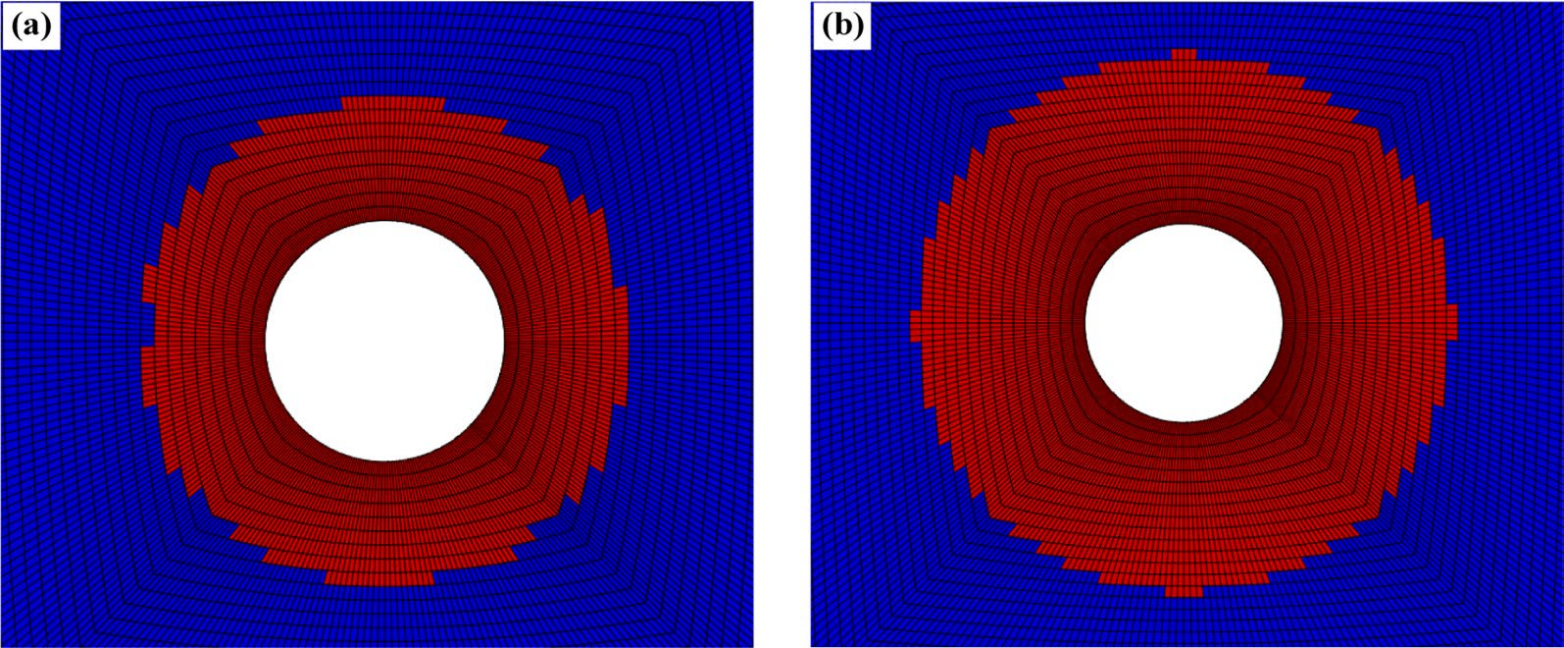
Comparison of the plastic zone between the (**a**)Mohr-Coulomb model and the (**b**) proposed model.

**Fig. 6:**
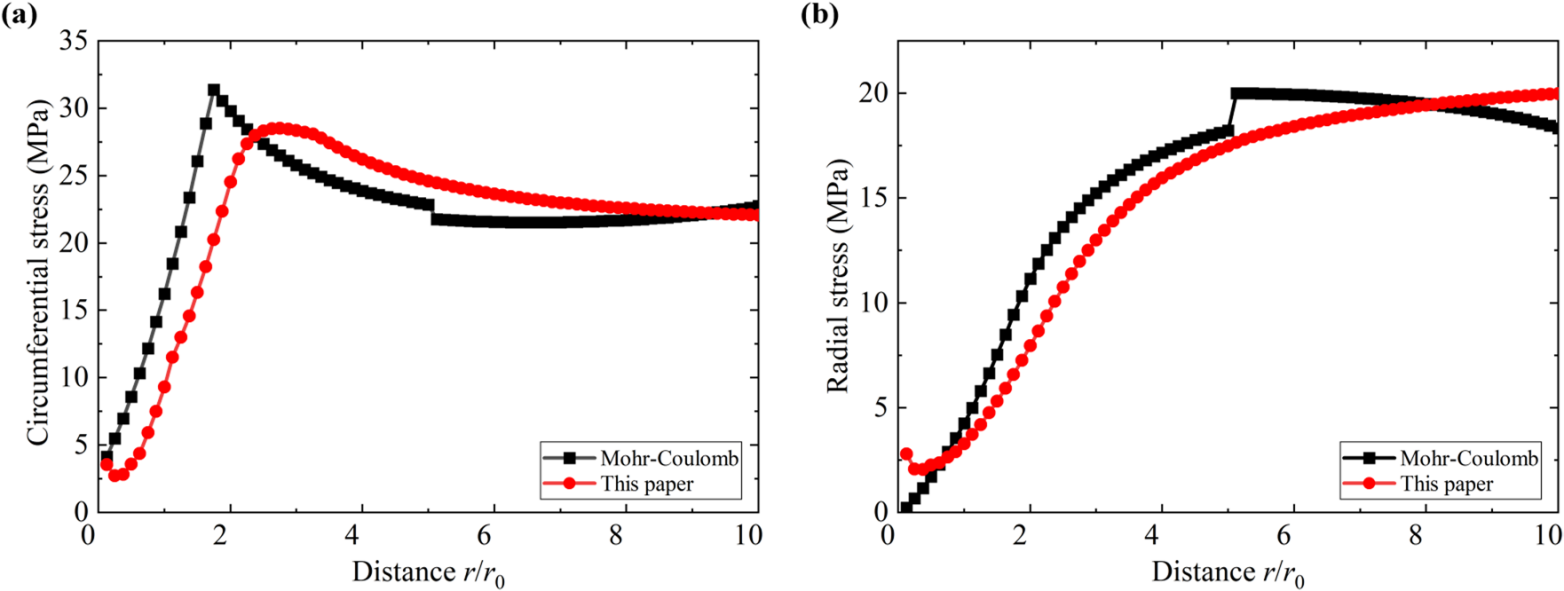
Comparison of the (**a**) circumferential stress and the (**b**) radial stress.

Figures 5 and 6 show comparisons of the plastic zones around the tunnel and the distribution patterns of maximum and minimum principal stresses in the surrounding rock predicted by the proposed model and the Mohr-Coulomb model. The figures indicate that the predicted plastic zones and stress distribution patterns around the tunnel are largely consistent between the two models. However, compared to the ideal elastic-plastic model based on the Mohr-Coulomb criterion, the model developed in this paper accounts for rock mass damage behavior. Consequently, it predicts a greater depth for the plastic zone and a larger depth for the loosened zone around the tunnel. In actual engineering projects, rock mass damage is unavoidable; failure to account for this damage behavior will lead to overestimation of the rock mass’s bearing capacity, resulting in an inadequately designed support scheme.

## Non-linear numerical simulation method of the interface

### Mathematical model of the thin-layer contact element

**Fig. 7:**
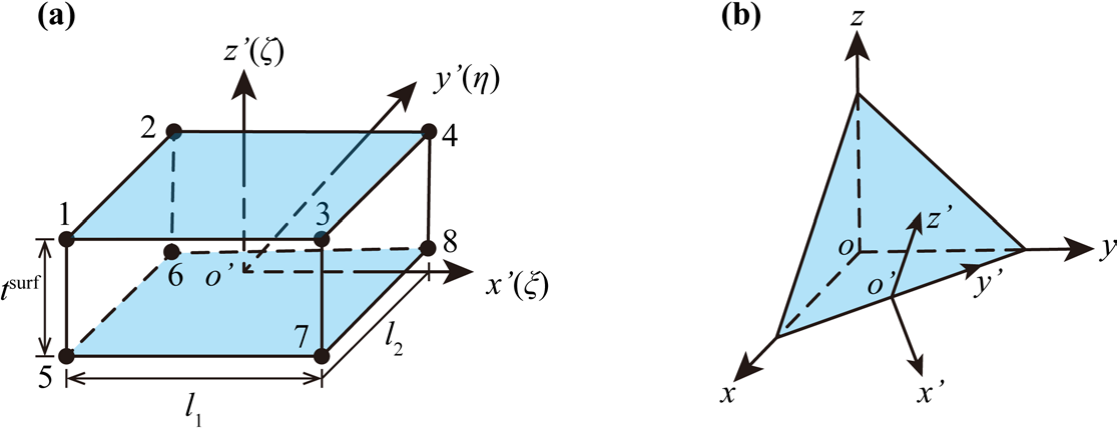
Mathematical model of the thin-layer contact element: (**a**) nodes’ arrangement and the local coordinate system and (**b**) the relationship between local and global coordinate systems.

Based on the Desai thin-layer contact element^30^ and referring to the research results of X.W. Wang et al.^34^, an 8-node thin-layer contact element shown in Fig. 7 is used to simulate the contact surface.

For each contact element, a local coordinate system as shown in Fig. 7(a) is established, and the contact element is divided into upper and lower planes along the direction normal to the contact surface, in which the upper plane is composed of nodes 1, 2, 3, and 4, and the lower plane is composed of nodes 5, 6, 7, and 8. The normal mechanical behavior of the contact element is described by normal stress and normal strain, and the tangential mechanical behavior of the contact element is described by tangential stress and shear strain. Under the local coordinate system, the relative displacement of any two corresponding nodes on the upper and lower planes of the contact element can be expressed as

12 $$\Delta {\mathbf{U}^{\prime}}={\left[ {\begin{array}{*{20}{c}} {u_{{{\text{top}}}}^{\prime } - u_{{{\text{bottom}}}}^{\prime },}&{v_{{{\text{top}}}}^{\prime } - v_{{{\text{bottom}}}}^{\prime },}&{w_{{{\text{top}}}}^{\prime } - w_{{{\text{bottom}}}}^{\prime }} \end{array}} \right]^{\text{T}}}$$

where: $u^{\prime},v^{\prime},w^{\prime}$ are the displacements in the local coordinate system; subscripts “top” and “bottom” correspond to the upper and lower planes, respectively. According to the finite element shape function interpolation theory, we have

13 $$\Delta {{\mathbf{U}}^{'}}=\left\{ {\begin{array}{*{20}{c}} {\sum\limits_{{i=1}}^{4} {{{\bar {N}}_i}(u_{i}^{\prime } - u_{{i+4}}^{\prime })} } \\ {\sum\limits_{{i=1}}^{4} {{{\bar {N}}_i}(v_{i}^{\prime } - v_{{i+4}}^{\prime })} } \\ {\sum\limits_{{i=1}}^{4} {{{\bar {N}}_i}(w_{i}^{\prime } - w_{{i+4}}^{\prime })} } \end{array}} \right\}={{\mathbf{N}}^{{\text{surf}}}}\left\{ {\begin{array}{*{20}{c}} {u_{1}^{\prime }} \\ {v_{1}^{\prime }} \\ {w_{1}^{\prime }} \\ \vdots \\ {u_{8}^{\prime }} \\ {v_{8}^{\prime }} \\ {w_{8}^{\prime }} \end{array}} \right\}={{\mathbf{N}}^{{\text{surf}}}}{{\mathbf{\delta }}^{'}}$$

where: ${\bar {N}_i}=0.25 \cdot (1+{\xi _i}\xi )(1+{\eta _i}\eta ),{\text{ }}i=1,2,3,4$; ${{\mathbf{N}}^{{\text{surf}}}}$is the shape function matrix, whose expression is

14 $${{\mathbf{N}}^{{\text{surf}}}}=\left[ {\begin{array}{*{20}{c}} {{{\bar {N}}_1}}&0&0& \cdots &{{{\bar {N}}_4}}&0&0&{ - {{\bar {N}}_1}}&0&0& \cdots &{ - {{\bar {N}}_4}}&0&0 \\ 0&{{{\bar {N}}_1}}&0& \cdots &0&{{{\bar {N}}_4}}&0&0&{ - {{\bar {N}}_1}}&0& \cdots &0&{ - {{\bar {N}}_4}}&0 \\ 0&0&{{{\bar {N}}_1}}& \cdots &0&0&{{{\bar {N}}_4}}&0&0&{ - {{\bar {N}}_1}}& \cdots &0&0&{ - {{\bar {N}}_4}} \end{array}} \right]$$

Assuming that the thickness of the contact element is ${t^{{\text{surf}}}}$, the strain component at any point of the contact element can be expressed as the ratio of the relative displacement of the upper and lower planes under the local coordinate system to the thickness of the element, i.e.

15 $${{\mathbf{\varepsilon }}^{{\text{surf}}}}=\left\{ {\begin{array}{*{20}{c}} {{\gamma _{x'}}} \\ {{\gamma _{y{'}}}} \\ {{\varepsilon _{z{'}}}} \end{array}} \right\}=\frac{1}{{{t^{{\text{surf}}}}}}\left\{ {\begin{array}{*{20}{c}} {\Delta {u^{'}}} \\ {\Delta {v^{'}}} \\ {\Delta {w^{'}}} \end{array}} \right\}=\frac{1}{{{t^{{\text{surf}}}}}}{{\mathbf{N}}^{{\text{surf}}}}{{\mathbf{\delta }}^{'}}$$

It should be noted that the value of the element thickness has a significant impact on the calculation results. According to the research by G.N. Pande and K.G. Sharma^35^, the element thickness can be taken as

16 $${t^{{\text{surf}}}}=\frac{{\hbox{max} ({l_1},{l_2})}}{{AR}}$$

where:$AR$is the aspect ratio, C.S. Desai et al.^30^ suggested that the value should not be less than 100.

As shown in Fig. 7(b), due to the difference between the local coordinate system and the global coordinate system, in order to integrate the contact element stiffness matrix into the global stiffness matrix, the element strain needs to be represented by the node displacement in the global coordinate system. Assuming the node displacement vector in the global coordinate system is $${\mathbf{\delta }}={\left[ {\begin{array}{*{20}{c}} {{u_1},}&{{v_1},}&{{w_1},}& \cdots &{{u_8},}&{{v_8},}&{{w_8}} \end{array}} \right]^{\text{T}}}$$, then the node displacement vector in the local coordinate system is ${\mathbf{\delta }}^{'} ={{\mathbf{T}}_0}{\mathbf{\delta }}$, where ${{\mathbf{T}}_0}$ is the coordinate transformation matrix, which can be expressed as

17 $${{\mathbf{T}}_0}=\left[ {\begin{array}{*{20}{c}} {{{\mathbf{\beta }}_0}}&{}&{}&{} \\ {}&{{{\mathbf{\beta }}_0}}&{}&{} \\ {}&{}& \ddots &{} \\ {}&{}&{}&{{{\mathbf{\beta }}_0}} \end{array}} \right],{\text{ }}{{\mathbf{\beta }}_0}=\left[ {\begin{array}{*{20}{c}} {{l_1}}&{{m_1}}&{{n_1}} \\ {{l_2}}&{{m_2}}&{{n_2}} \\ {{l_3}}&{{m_3}}&{{n_3}} \end{array}} \right]$$

where: ${l_i},{\text{ }}{m_i},{\text{ }}{z_i}$ represent the directional cosine of the *i*-axis of the local coordinate system *o’-x’y’z’* in the direction of the *x*, *y*, *z* axes of the global coordinate system *o-xyz*, respectively. Thus the element strain matrix can be obtained as ${{\mathbf{B}}^{{\text{surf}}}}={{{{\mathbf{N}}^{{\text{surf}}}}{{\mathbf{T}}_0}} \mathord{\left/ {\vphantom {{{{\mathbf{N}}^{{\text{surf}}}}{{\mathbf{T}}_0}} {{t^{{\text{surf}}}}}}} \right. \kern-0pt} {{t^{{\text{surf}}}}}}$, according to the virtual work principle, the element stiffness matrix of the contact element is

18 $${{\mathbf{K}}^{{\text{surf}}}}={t^{{\text{surf}}}} \cdot \int_{{ - 1}}^{1} {\int_{{ - 1}}^{1} {{{\left( {{{\mathbf{B}}^{{\text{surf}}}}} \right)}^{\text{T}}}} } {\mathbf{D}}_{{\text{e}}}^{{{\text{surf}}}}{{\mathbf{B}}^{{\text{surf}}}}\left| {\mathbf{J}} \right|{\text{d}}\xi {\text{d}}\eta$$

where: ${\mathbf{D}}_{{\text{e}}}^{{{\text{surf}}}}$ is the elastic matrix of the contact element, whose expression is^36^

19 $${\mathbf{D}}_{{\text{e}}}^{{{\text{surf}}}}=\left[ {\begin{array}{*{20}{c}} {{G_{x'}}}&0&{ - {E_{z'}}{\lambda _{x'}}\tan {\alpha ^{{\text{surf}}}}} \\ 0&{{G_{y'}}}&{ - {E_{z'}}{\lambda _{y'}}\tan {\alpha ^{{\text{surf}}}}} \\ { - {E_{z'}}{\lambda _{x'}}\tan {\alpha ^{{\text{surf}}}}}&{ - {E_{z'}}{\lambda _{y'}}\tan {\alpha ^{{\text{surf}}}}}&{{E_{z'}}} \end{array}} \right]$$

where: ${\alpha ^{{\text{surf}}}}$ is the dilatation angle of the contact surface; ${G_{x'}}$ and ${G_{y'}}$ is the shear modulus along the tangential direction of the contact surface; ${E_{z'}}$ is the Young’s modulus along the normal direction of the contact surface; ${\lambda _{x'}}$ and ${\lambda _{y'}}$ are the shear expansion/contraction control parameters, when ${\gamma _{x'}}{\text{d}}({\gamma _{x'}})>0{\text{ }}({\gamma _{y'}}{\text{d}}({\gamma _{y'}})>0)$, ${\lambda _{x'}}=1{\text{ }}({\lambda _{y'}}=1)$, on the contrary, ${\lambda _{x'}}= - 1{\text{ }}({\lambda _{y'}}= - 1)$. ${G_{x'}}$, ${G_{y'}}$ and ${E_{z'}}$can be expressed as the product of the shear stiffness ${K_{x'}},{K_{y'}}$, normal stiffness ${K_{z'}}$, and the thickness of the contact element, i.e. ${G_{x'}}={K_{x'}} \cdot {t^{{\text{surf}}}}$, ${G_{y'}}={K_{y'}} \cdot {t^{{\text{surf}}}}$, and ${E_{z'}}={K_{z'}} \cdot {t^{{\text{surf}}}}$.

### Failure modes and peak shear strength model of the structural surface

#### Failure modes

**Fig. 8:**
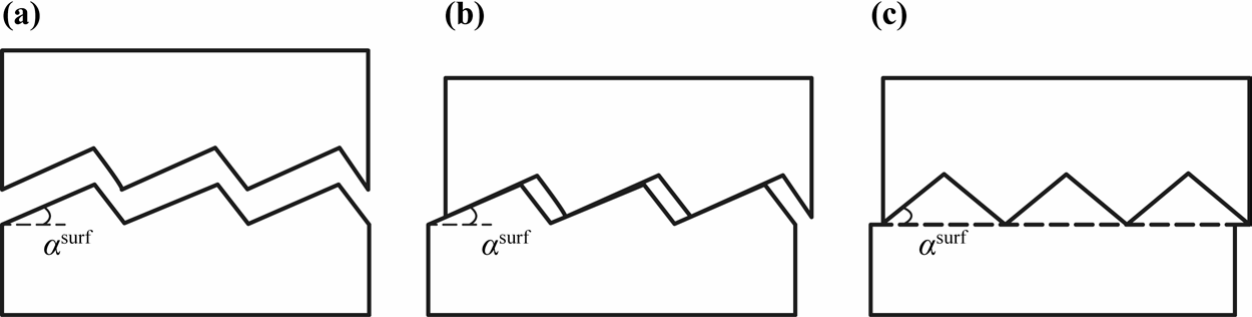
Failure modes of the contact surface: (**a**) disengagement damage, (**b**) abrasion damage, and (**c**) snip damage.

Using artificially prepared specimens of regular serrated concrete joints, H.B. Li et al.^37^ investigated the joint’s failure modes, stiffness characteristics, and their relationship with the undulation angle, shear deformation rate, and normal stress. X.F. Li et al.^38^ conducted indoor direct shear tests on artificial concrete joints with different undulation angles to investigate the failure modes and stress-strain relationship. The results of all the above scholars show that there are three different damage patterns for the contact surface, i.e., disengagement damage, abrasion damage, and snip damage (see Fig. 8).

The disengagement damage of the contact surface is due to the normal tensile stress exceeding the tensile strength of the contact surface, the abrasion damage and snip damage of the contact surface are both caused by the shear stress, and the shear damage modes of the contact surface are closely related to the undulation angle and normal stress. When the undulation angle is small or the normal stress is small, under the action of shear stress, relative sliding occurs along the direction of the contact surface bulge, and the contact surface occurs wear and tear damage, at this time, the contact surface has an obvious climbing effect. When the undulation angle is large or the normal stress is large, the bulge on the contact surface is directly sheared off, at this time, there is an obvious nibbling fracture zone in the shear stress-relative deformation curve.

For the contact surface damaged by abrasion, its shear stress-deformation curve can be divided into two stages, i.e., the climbing zone and slip zone, there is no obvious nibbling zone, and the gap between its peak shear stress and residual shear stress is relatively small. For the contact surface damaged by snip, its shear stress-deformation curve can be divided into three stages, i.e., the climbing zone, nibbling zone, and slip zone. Its residual shear strength is significantly lower than the peak shear strength^37^.

#### Peak shear strength model

**Fig. 9:**
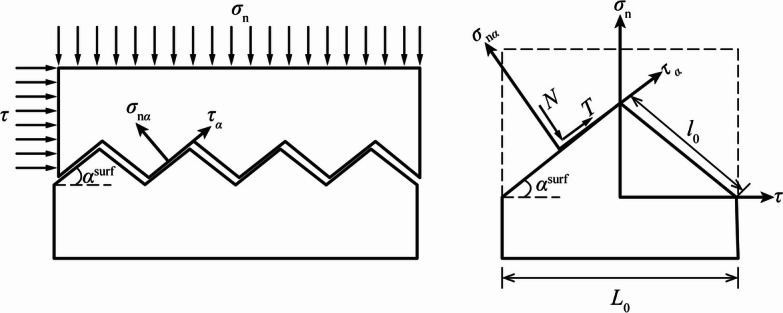
Force analysis schematic of the regular contact surface.

The peak shear strength is an important mechanical parameter to characterize the shear mechanical properties of the contact surface, and the peak shear strength is closely related to the failure modes of the contact surface. The force analysis schematic of the regular contact surface is shown in Fig. 9, and the peak shear strengths of the contact surface under different failure modes are derived as follows.

##### Abrasion damage

The abrasion damage mechanism of the contact surface is that the relative sliding occurs along the contact surface bulge under the action of shear stress. In this case, the peak shear strength is related to the cohesion and friction coefficient of the surface of the bulge. As shown in Fig. 9, it is assumed that the upper disk portion of the contact surface moves to the right relative to the lower disk portion under an external force.

Taking one of the individual undulations for analysis, it is obvious that the left and right sides of the rough bump are in compressive shear and tensile shear states, respectively, and the ultimate shear strength of the left side is much larger than that of the right side, and when the right side undergoes shear damage, the left side can continue to withstand the shear load^39^. Therefore, the peak shear strength of the contact surface is determined by the left side of the rough bump, and when the shear stress reaches the shear strength, the force equilibrium equations for a single bump are established as follows.

20 $$\left\{ {\begin{array}{*{20}{c}} {T\cos ({\alpha ^{{\text{surf}}}}) - N\sin ({\alpha ^{{\text{surf}}}})={\tau _{\text{p}}}{L_0}} \\ {T\sin ({\alpha ^{{\text{surf}}}})+N\cos ({\alpha ^{{\text{surf}}}})={\sigma _{\text{n}}}{L_0}} \end{array}} \right.$$

where: *T* and *N* are the shear force and compression force of the left side of a single bump, respectively $T=N\tan ({\varphi _{\text{b}}})+{c_{\text{b}}}{l_0}$; ${\tau _{\text{p}}}$ and ${\sigma _{\text{n}}}$ are the peak shear strength and normal stress at the contact surface; ${\varphi _{\text{b}}}$ and ${c_{\text{b}}}$ are the cohesion and the internal friction angle of the surface of the bulge; ${l_0}$ and ${L_0}$ are the geometry of the bulge. Elimination of *T* and *N* from Eq. (20) gives the peak shear strength of the contact surface in the abrasion damage mode as

21 $${\tau _{\text{p}}}= - {\sigma _{\text{n}}}\tan ({\varphi _{\text{b}}}+{\alpha ^{{\text{surf}}}})+\frac{{{c_{\text{b}}}}}{{2\cos ({\alpha ^{{\text{surf}}}})\left[ {\cos ({\alpha ^{{\text{surf}}}}) - \sin ({\alpha ^{{\text{surf}}}})\tan ({\varphi _{\text{b}}})} \right]}}$$

##### Snip damage

The snip damage mechanism of the contact surface is that under the action of shear force, the contact surface shear stress exceeds the shear strength of the bump, resulting in fracture inside the bump, so that the contact surface undergoes shear damage along the plane of the root of the bump. At this point, the shear strength is related to the cohesion and internal friction angle of the rock mass itself, which can be expressed as^39^

22 $${\tau _{\text{p}}}= - {\sigma _{\text{n}}}\tan ({\varphi _{\text{r}}})+{c_{\text{r}}}$$

where: ${\varphi _{\text{r}}}$ and ${c_{\text{r}}}$ are the internal friction angle and cohesion of the rock mass.

For a given contact surface, the failure mode and peak shear stress are determined by the normal stress. Let Eq. (21) equal to Eq. (22), then the critical normal stress is

23 $${\sigma _{{\text{n,c}}}}=\frac{{{c_{\text{r}}} - {{{c_{\text{b}}}} \mathord{\left/ {\vphantom {{{c_{\text{b}}}} {2\cos ({\alpha ^{{\text{surf}}}})\left[ {\cos ({\alpha ^{{\text{surf}}}}) - \sin ({\alpha ^{{\text{surf}}}})\tan ({\varphi _{\text{b}}})} \right]}}} \right. \kern-0pt} {2\cos ({\alpha ^{{\text{surf}}}})\left[ {\cos ({\alpha ^{{\text{surf}}}}) - \sin ({\alpha ^{{\text{surf}}}})\tan ({\varphi _{\text{b}}})} \right]}}}}{{\tan ({\varphi _{\text{r}}}) - \tan ({\varphi _{\text{b}}}+{\alpha ^{{\text{surf}}}})}}$$

Thus, the peak shear strength of the contact surface is

24 $${\tau _{\text{p}}}=\left\{ {\begin{array}{*{20}{c}} \begin{gathered} - {\sigma _{\text{n}}}\tan ({\varphi _{\text{b}}}+{\alpha ^{{\text{surf}}}})+ \hfill \\ \frac{{{c_{\text{b}}}}}{{2\cos ({\alpha ^{{\text{surf}}}})\left[ {\cos ({\alpha ^{{\text{surf}}}}) - \sin ({\alpha ^{{\text{surf}}}})\tan ({\varphi _{\text{b}}})} \right]}}, \hfill \\ \end{gathered} &{{\sigma _{\text{n}}}>{\sigma _{{\text{n,c}}}}({\text{abrasion damage)}}} \\ { - {\sigma _{\text{n}}}\tan ({\varphi _{\text{r}}})+{c_{\text{r}}},}&{{\sigma _{\text{n}}} \leqslant {\sigma _{{\text{n,c}}}}({\text{snip damage}})} \end{array}} \right.$$

When the shear stress reaches the peak shear strength, the contact surface undergoes shear damage, and its shear stress gradually decreases to the residual shear strength with the increase of the relative shear displacement. The residual strength of the contact surface is controlled by the normal stress and internal friction angle, which can be expressed as

25 $${\tau _{\text{r}}}=\left\{ {\begin{array}{*{20}{c}} { - {\sigma _{\text{n}}}\tan ({\varphi _{\text{b}}}+{\alpha ^{{\text{surf}}}}),}&{{\sigma _{\text{n}}}>{\sigma _{{\text{n,c}}}}({\text{abrasion damage}})} \\ { - {\sigma _{\text{n}}}\tan ({\varphi _{\text{r}}}),}&{{\sigma _{\text{n}}} \leqslant {\sigma _{{\text{n,c}}}}({\text{snip damage}})} \end{array}} \right.$$

### Non-linear constitutive relationship of the interface

#### Normal non-linear mechanical behavior and its constitutive relationship

When the normal tensile stress of the contact surface exceeds the tensile strength, the contact surface is disengaged, at which time the mechanical action of the contact surface will be completely lost, so the elastic-brittle model is used in this paper to describe the normal tensile behavior of the contact surface. For the normal compression behavior of the contact surface, S.C. Bandis et al.^51^ proposed the following hyperbolic model based on numerous experimental data

26 $$K_{z{'}}={K_{z{'}0}}{\left( {1 - \frac{{\left| {\Delta w^{'}} \right|}}{{{V_{\text{m}}}}}} \right)^{ - 2}}$$

where: ${K_{z'0}}$ is the initial normal stiffness; ${V_{\text{m}}}$ is the maximum closure of the contact element, which generally can be taken as the element’s thickness^40^. From Eq. (26), it can be seen that with the increase of the compression relative displacement of the contact element, the normal compression stiffness gradually increases, and when the normal relative displacement is close to the element’s thickness, the normal stiffness tends to infinity, so the model can effectively avoid the element embedding problem.

#### Tangential non-linear mechanical behavior and its constitutive relationship

The tangential nonlinear mechanical behavior of the contact surface is divided into two deformation stages, i.e., the elastic deformation stage before the damage of the contact surface and the slip deformation stage after the damage. The shear damage criterion of the two-dimensional contact surface in the local coordinate system is

$${F^{{\text{surf}}}}=\sqrt {{{({\tau _{x'}})}^2}+{{({\tau _{y'}})}^2}} - {\tau _{\text{p}}} \geqslant 0$$.

For the convenience of application, the nonlinear deformation behavior of the contact surface in the pre-peak stage is not considered in this paper, and it is assumed that the contact surface satisfies the linear-elastic constitutive relationship in this stage. When the shear stress exceeds the peak shear strength, the contact surface undergoes damage and enters a post-peak softening stage, where the shear stress is gradually reduced to the residual shear strength.

The experimental results of G. Grassellia et al.^41^ and H.S. Lee et al.^5^ show that the post-peak softening curve is close to a hyperbolic curve. Based on this finding, and referring to the one-dimensional contact surface shear softening curve proposed by X.J. Yin et al.^9^, the following two-dimensional contact surface post-peak softening curve was proposed

27 $$\tau ={\tau _{\text{r}}}+({\tau _{\text{p}}} - {\tau _{\text{r}}}){\left( {\frac{{\left| {\Delta {s_{\text{p}}}} \right|}}{{\sqrt {{{(\Delta {u^{'}})}^{2}}+{{(\Delta {v^{'}})}^2}} }}} \right)^\eta }$$

where: ${\tau _{\text{p}}}$ and ${\tau _{\text{r}}}$ are the peak shear strength and residual shear strength, which can be calculated by Eqs. (24)-(25); $\Delta {s_{\text{p}}}$ is the relative displacement corresponding to the peak shear strength; $\eta$ is a constant to characterize the softening velocity of the contact surface, which can be obtained by fitting the experimental data. Equation (27) describes the post-peak softening curve along the direction of the maximum shear displacement of the contact surface, and the post-peak shear stresses along both *x’* and *y’* directions in the local coordinate system can be calculated by

28 $${\tau _{x{'}}}=\tau {{\left| {\Delta {u^{'}}} \right|} \mathord{\left/ {\vphantom {{\left| {\Delta {u^{'}}} \right|} {\sqrt {{{(\Delta {u^{'}})}^2}+{{(\Delta {v^{'}})}^2}} }}} \right. \kern-0pt} {\sqrt {{{(\Delta {u^{'}})}^2}+{{(\Delta {v^{'}})}^2}} }},{\text{ }}{\tau _{y{'}}}=\tau {{\left| {\Delta {v^{'}}} \right|} \mathord{\left/ {\vphantom {{\left| {\Delta {v^{'}}} \right|} {\sqrt {{{(\Delta {u^{'}})}^2}+{{(\Delta {v^{'}})}^2}} }}} \right. \kern-0pt} {\sqrt {{{(\Delta {u^{'}})}^2}+{{(\Delta {v^{'}})}^2}} }}$$

#### Shear expansion/contraction phenomena and shear expansion angle degradation effect

Due to the presence of rough bumps on the contact surface, the shear displacement will affect the normal displacement during shear deformation, i.e., there is a shear expansion/shear contraction phenomenon. The shear expansion/shear contraction phenomenon at the contact surface can be reflected by introducing a normal and tangential coupling term in the constitutive relationship, i.e., the term on the non-diagonal in Eq. (19).

During the deformation process of the contact surface, the rough bumps will be gradually worn out, it will result in the degradation of the shear expansion angle, which can be described by an exponential function^4^

29 $${\alpha ^{{\text{surf}}}}=\alpha _{{\text{0}}}^{{{\text{surf}}}}\exp ( - {\beta ^{{\text{surf}}}}{W^{{\text{surf}}}})$$

where: $\alpha _{{\text{0}}}^{{{\text{surf}}}}$is the initial shear expansion angle; ${\beta ^{{\text{surf}}}}$ is a parameter to control the degradation rate of the shear expansion angle; ${W^{{\text{surf}}}}$is the plastic shear work, $${\text{d}}{W^{{\text{surf}}}}=\left[ {\begin{array}{*{20}{c}} {{\tau _{x'}},}&{{\tau _{y'}}} \end{array}} \right] \cdot {\left[ {\begin{array}{*{20}{c}} {{\text{d}}(\Delta u_{{\text{p}}}^{\prime }),}&{{\text{d}}(\Delta v_{{\text{p}}}^{\prime })} \end{array}} \right]^{\text{T}}}$$. ${\beta ^{{\text{surf}}}}$ can be determined by^42^

30 $${\beta ^{{\text{surf}}}}=0.141\alpha _{{\text{0}}}^{{{\text{surf}}}} \cdot \frac{{{\sigma _{z'}}}}{{{\sigma _{\text{c}}}}}$$

where: ${\sigma _{\text{c}}}$ is the compressive strength of the rock mass.

### Verification of the non-linear constitutive relationship of the interface

#### Comparisons with the direct shear test data for rock-like joints obtained by X.F. Li et al.^38^

**Fig. 10:**
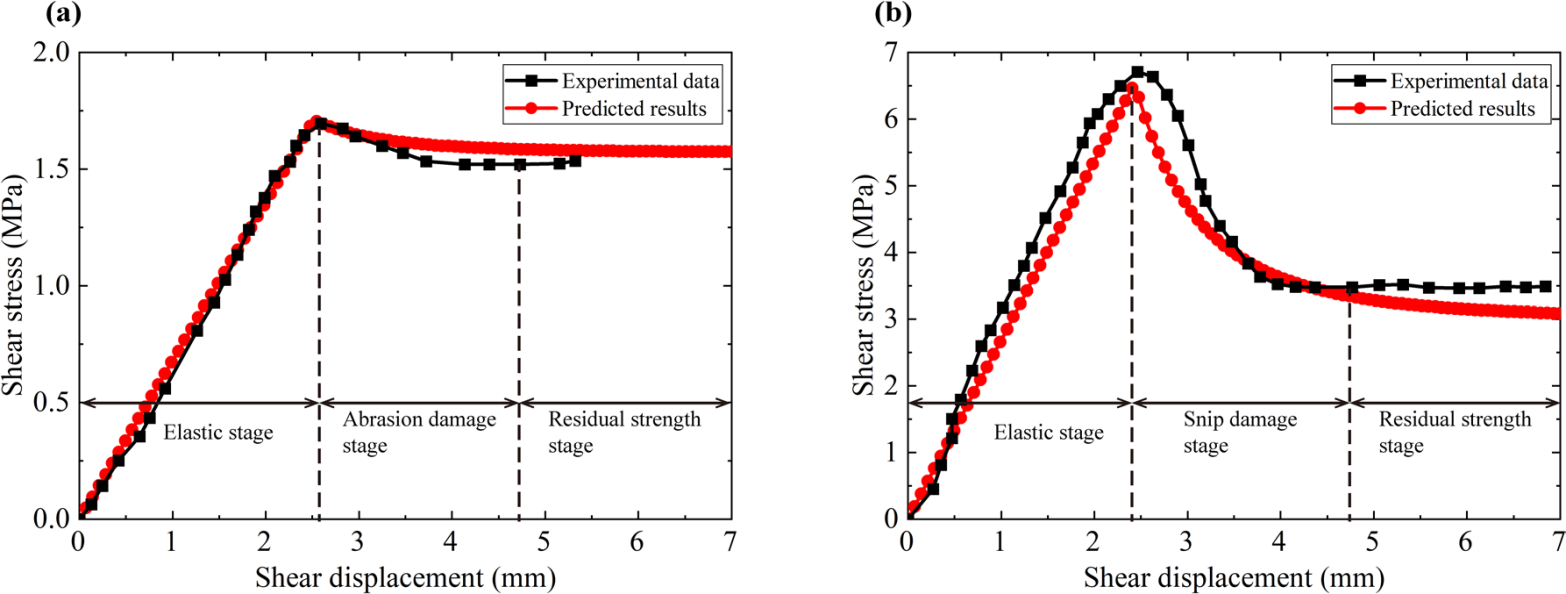
Comparisons between the predicted results of the proposed model and the test data obtained by X.F. Li^38^. (**a**) Specimen 1 and (**b**) specimen 2.

X.F. Li et al.^38^ conducted direct shear tests on two sets of uniformly serrated joint specimens and obtained joint shear stress-shear displacement curves (see Fig. 10). Specimen 1: normal stress of 1 MPa, initial shear expansion angle of 15°; Specimen 2: normal stress of 3 MPa, initial shear expansion angle of 45°. The mechanical parameters of the structural surface are tabled in Table 1, and the comparisons between the model prediction results and the test data are shown in Fig. 10. As shown in Fig. 10(a), when the undulation angle and normal stress of the structural surface are small, the structural surface undergoes abrasion damage, there is no obvious “nibbling zone”, and the residual shear strength of the structural surface does not differ much from the peak shear strength. When the undulation angle and normal stress of the structural surface are large, the structural surface undergoes snap damage, with a clear “nibbling zone”, at which time the residual shear strength of the structural surface is significantly lower than the peak shear strength (see Fig. 10 [b]). The model prediction results of the two groups of specimens are basically consistent with the experimental data, indicating that the proposed model can reasonably simulate the abrasion damage and snap damage of the structural surface and correctly predict its post-peak softening behavior.

**Table 1:** Mechanical parameters of the structural surface.

Specimens	Initial shear stiffness (MPa/m)	Inner friction angle of the rock mass $\:{\phi\:}_{\text{r}}$ (°)	Cohesion of the rock mass $\:{c}_{\text{r}}$ (MPa)	Inner friction of the structural surface $\:{\phi\:}_{\text{b}}$ (°)	Cohesion of the structural surface $\:{c}_{\text{b}}$ (MPa)	Initial dilatancy angle $\:{\alpha\:}^{\text{s}\text{u}\text{r}\text{f}}$ (°)	Softening velocity constant $\:\eta\:$
Specimen 1	680.00	45.00	3.50	42.50	0.20	15.00	3.50
Specimen 2	2690.00	45.00	3.50	42.50	0.20	45.00	3.50

#### Comparisons with the direct shear test data for artificial surface obtained by S.C. Bandis et al.^51^

**Fig. 11:**
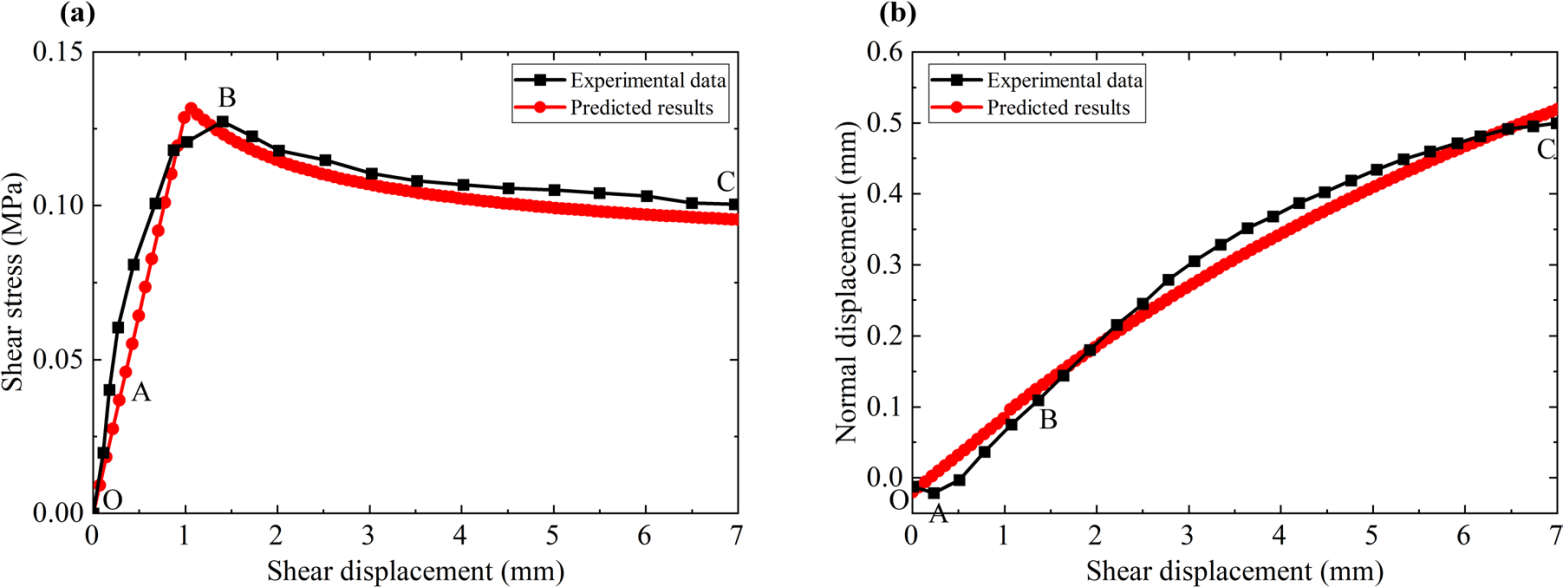
Comparisons between the predicted results of the proposed model and the test data obtained by S.C. Bandis et al.^51^ (**a**) Shear stress-shear displacement curve and (**b**) normal displacement-shear displacement curve.

S.C. Bandis et al.^51^ conducted a direct shear test using an artificial structural surface and obtained the shear stress-shear displacement curve and the normal displacement-shear displacement curve. The normal load is 0.9 MPa, the initial shear stiffness is 130 MPa/m, the compression strength of the rock mass is −2.0 MPa, the inner friction angle of the structural surface is 35.0°, the cohesion of the structural surface is 0.1 MPa, the initial dilatancy angle is 6°, the softening velocity constant is 0.6. Comparisons between the model prediction results and the test data are shown in Fig. 11.

As shown in Fig. 11(a), at the beginning of loading, the structural surface is in the elastic deformation stage, and the shear stress increases linearly with the increase of shear displacement (OA segment). Subsequently, the shear stiffness of the structural surface gradually decreases, showing obvious nonlinear deformation characteristics until the peak strength is reached (AB segment). When the shear stress reaches the peak strength, abrasion damage occurs on the structural surface and the shear stress gradually decreases until the residual shear strength (BC segment) is reached. The model predictions are in general agreement with the experimental data, but the shear stress-shear displacement curve at the pre-peak stage is slightly different from the experimental data since it is assumed that the structural surface satisfies the linear elastic constitutive relationship at the pre-peak stage.

Figure 11(b) shows that the shear expansion curve of the structural face can be divided into the following typical stages. At the beginning of loading, the normal displacement is slightly reduced, but it lasts for a short time (OA segment). Subsequently, due to the shear expansion effect, the normal displacement of the structural surface increases linearly with the shear displacement (AB segment). Finally, due to the abrasion effect, the shear expansion angle gradually degrades, and the shear expansion curve gradually tends to flatten (BC segment). The shear expansion curve predicted by the model is basically consistent with the test data.

## Stability evaluation method of the surrounding rock

### Yielding approaching index and its calculation formula

The concept of yielding approaching index was first proposed by H. Zhou^43^ in 2005, and redefined by L.Y. Gao^44^ in 2016, i.e., keeping the Lode angle constant in the $\pi$ plane, the ratio of the distance from the stress point to the yield surface and the distance from the origin of the $\pi$ plane to the yield surface along the direction of the line connecting the stress point and the origin of the $\pi$ plane.

As shown in Fig. 1(b), the yielding approach index is $YAI={A_0}{A_1}/O{A_1}$, and its phase complement parameter is $\omega _{{YAI}}^{{\text{r}}}=O{A_0}/O{A_1}$. According to the elastic-plastic theory, $O{A_0}=\sqrt {2{{\left. {\bar {J}_{{\text{2}}}^{{\text{r}}}} \right|}_{{A_0}}}}$, $O{A_1}=\sqrt {2{{\left. {\bar {J}_{{\text{2}}}^{{\text{r}}}} \right|}_{{A_1}}}}$. Thus the phase complement parameter of the yielding approach index is $\omega _{{YAI}}^{{\text{r}}}=\sqrt {{{{{\left. {\bar {J}_{{\text{2}}}^{{\text{r}}}} \right|}_{{A_0}}}} \mathord{\left/ {\vphantom {{{{\left. {\bar {J}_{{\text{2}}}^{{\text{r}}}} \right|}_{{A_0}}}} {{{\left. {\bar {J}_{{\text{2}}}^{{\text{r}}}} \right|}_{{A_1}}}}}} \right. \kern-0pt} {{{\left. {\bar {J}_{{\text{2}}}^{{\text{r}}}} \right|}_{{A_1}}}}}}$. Among them, ${\left. {\bar {J}_{{\text{2}}}^{{\text{r}}}} \right|_{{A_0}}}$ and ${\left. {\bar {J}_{{\text{2}}}^{{\text{r}}}} \right|_{{A_1}}}$ are the second invariant of the deviatoric stress at point *A*_0_ and point *A*_1_. Keeping the Lode angle constant in the $\pi$ plane and making Eq. (6) zero, the expression of ${\left. {\bar {J}_{{\text{2}}}^{{\text{r}}}} \right|_{{A_1}}}$ is

31 $${\left. {\bar {J}_{{\text{2}}}^{{\text{r}}}} \right|_{{A_1}}}{\text{ = }}g{\left( \theta \right)^2} \cdot \left[ { - {\alpha _{\text{r}}}{{\left( {\bar {\sigma }_{{\text{m}}}^{{\text{r}}}+\frac{{{\beta _{\text{r}}}}}{{2{\alpha _{\text{r}}}}}} \right)}^2} - {\gamma _{\text{r}}}+\frac{{{\beta _{\text{r}}}^{2}}}{{4{\alpha _{\text{r}}}}}} \right]$$

Apparently, $0 \leqslant \omega _{{YAI}}^{{\text{r}}} \leqslant 1$, the larger the value of $\omega _{{YAI}}^{{\text{r}}}$, the more dangerous the rock element, $\omega _{{YAI}}^{{\text{r}}}$ is able to quantitatively evaluate the safety degree of the rock elements that are in the elastic zone.

### Element dangerous coefficient of the rock element

In order to quantitatively evaluate the safety degree of the element that enters the plastic state, based on the yielding approaching index, the following element dangerous coefficient is defined by introducing the damage coefficient of the rock element ${d^{\text{r}}}$

32 $$EDC=\left\{ {\begin{array}{*{20}{c}} {\omega _{{YAI}}^{{\text{r}}},}&{{F^{\text{r}}}({{{\mathbf{\bar {\sigma }}}}^{\text{r}}})<0} \\ {1+{d^{\text{r}}},}&{{F^{\text{r}}}({{{\mathbf{\bar {\sigma }}}}^{\text{r}}}) \geqslant 0} \end{array}} \right.{\text{ }}$$

Apparently, $0 \leqslant EDC \leqslant 2$, the larger the element dangerous coefficient is, the more dangerous the rock element is, and the element dangerous coefficient can be used to quantitatively evaluate the danger degree of the surrounding rock in different deformation stages.

### Element dangerous coefficient of the interface element

Similar to the rock element, the element dangerous coefficient of the interface element is defined as follows. Considering that the damage of the structural surface is mainly divided into disengagement damage and shear damage (abrasion damage and shear damage), the element dangerous coefficient should be defined by combining the two damage modes. When the contact surface is in the elastic deformation stage, its element dangerous coefficient is

33 $$EDC=\hbox{max} \left( {\frac{{{\sigma _{z'}}}}{{{f_{{\text{ts}}}}}},\frac{{\sqrt {{{({\tau _{x'}})}^2}+{{({\tau _{y'}})}^2}} }}{{{\tau _{\text{p}}}}}} \right)$$

where: ${f_{{\text{ts}}}}$ is the tensile strength of the contact surface. When the shear damage occurs, the definition of the element dangerous coefficient is shown in Eq. (34). When the normal stress on the contact surface exceeds the tensile strength, the contact surface is disengaged, and the contact surface completely loses the load-bearing role, at which time the element dangerous coefficient is 2.0.

34 $$EDC=2 - \frac{{\left| {\Delta {s_{\text{p}}}} \right|}}{{\sqrt {{{(\Delta {u^{'}})}^2}+{{(\Delta {v^{'}})}^2}} }}$$

In the evaluation of the overall stability of the tunnel, the plastic zone volume *V*_p_ (the cumulative value of the volumes of all plastic elements) is widely used by many scholars as a quantitative index^45^. Similarly, an index called *Total_EDC* (the cumulative value of all elements’ *EDC*) is defined to evaluate the overall disturbance degree of the tunnel. All of the abovementioned models and methods were embedded into a self-developed finite element calculation software^46^ to form a complete calculation and analysis method for the cross-fault hydraulic tunnels.

## Engineering example analyses

### Finite element model and calculation parameters

The Quanmutang Reservoir Project is a significant water conservancy project to alleviate the problem of water shortage in the Hunan Province of China. The Jiulongling Tunnel is located in its main canal, with the entrance pile number ZG18 + 354.2 and the exit pile number ZG37 + 616, and its total length is 19261.8 m. The cross-section of the tunnel is in the shape of a city gate, and the excavation size is width×height = 6.3 m×6.29 m. There is a large burial depth, poor surrounding rock conditions, and several geological faults at the ZG31 + 0 ~ ZG32 + 880 section, to maintain the stability of the tunnel, the following supporting measures were adopted: systematic mortar anchors with a spacing of 1.25 m, a row spacing of 1.5 m, a diameter of 22 mm, and a length of 3 m; 0.15 m-thick C20 shotcrete; “I16” steel sets with a spacing of 0.55 m.

**Fig. 12:**
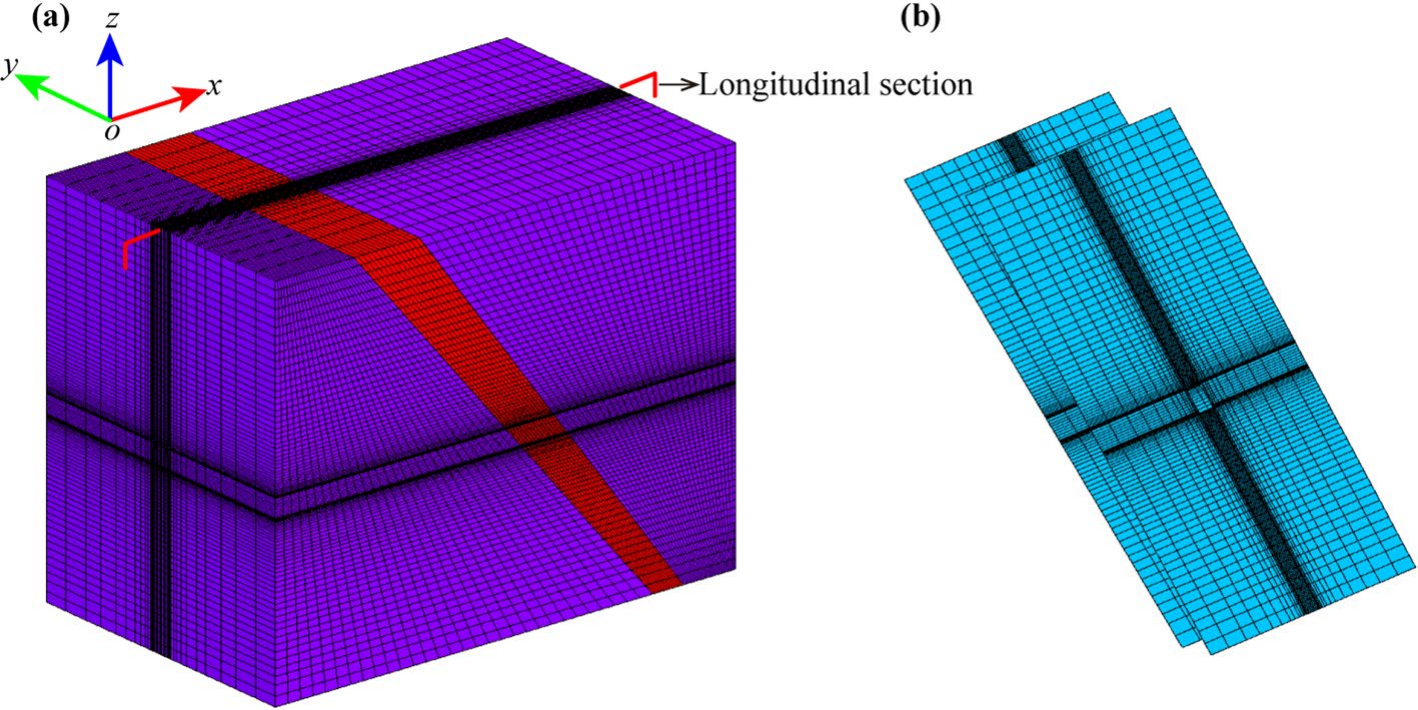
Finite element models. (**a**) Overall finite element model, and (**b**) contact elements’ model.

In order to conduct stability analysis for the surrounding rock of the tunnel, a finite element model consisting of a fault was established, as shown in Fig. 12. Among them, Fig. 12(a) is the overall finite element model, and the main rock mass within the model’s range is classified as Class IV. The fault is connected to the surrounding rock through the thin-layer contact element, the finite model of contact elements is shown in Fig. 12(b).

The basic mechanical parameters of the surrounding rock are tabulated in Table 2, and the expansion parameter is ${\alpha ^{\text{r}}}=0.3$, the ultimate damage coefficient is ${d^{{\text{ru}}}}=0.95$, and the damage constants are ${B_{\text{r}}}=300$, ${C_{\text{r}}}=1.0$. The Young’s modulus of the bolt is 200 GPa, the yielding strength is 400 MPa, and the ultimate strength is 560 MPa. The Young’s modulus of the steel set is 200 GPa, the yielding strength is 235 MPa, and the ultimate strength is 338 MPa. The anchor bolts were simulated through the method proposed by C. Kong et al.^47,48^, the steel sets were simulated by the implicit nonlinear simulation method^49^, and the shotcrete was simulated by the elastic damage model^50^.

**Table 2:** Mechanical parameters of the rock mass and shotcrete.

Materials	Young’s modulus (GPa)	Poisson’s ratio	Cohesion (MPa)	Inner friction angle (°)	Compression strength (MPa)	Tensile strength (MPa)
IV class surrounding rock	3.00	0.32	0.45	30.00	−20.0	——
Fault	0.40	0.40	0.08	21.80	−4.00	——
C20 shotcrete	25.50	0.17	——	40.00	−9.60	1.10

### Analyses of the distribution of the failure zone of the surrounding rock in the tunnel

**Fig. 13:**
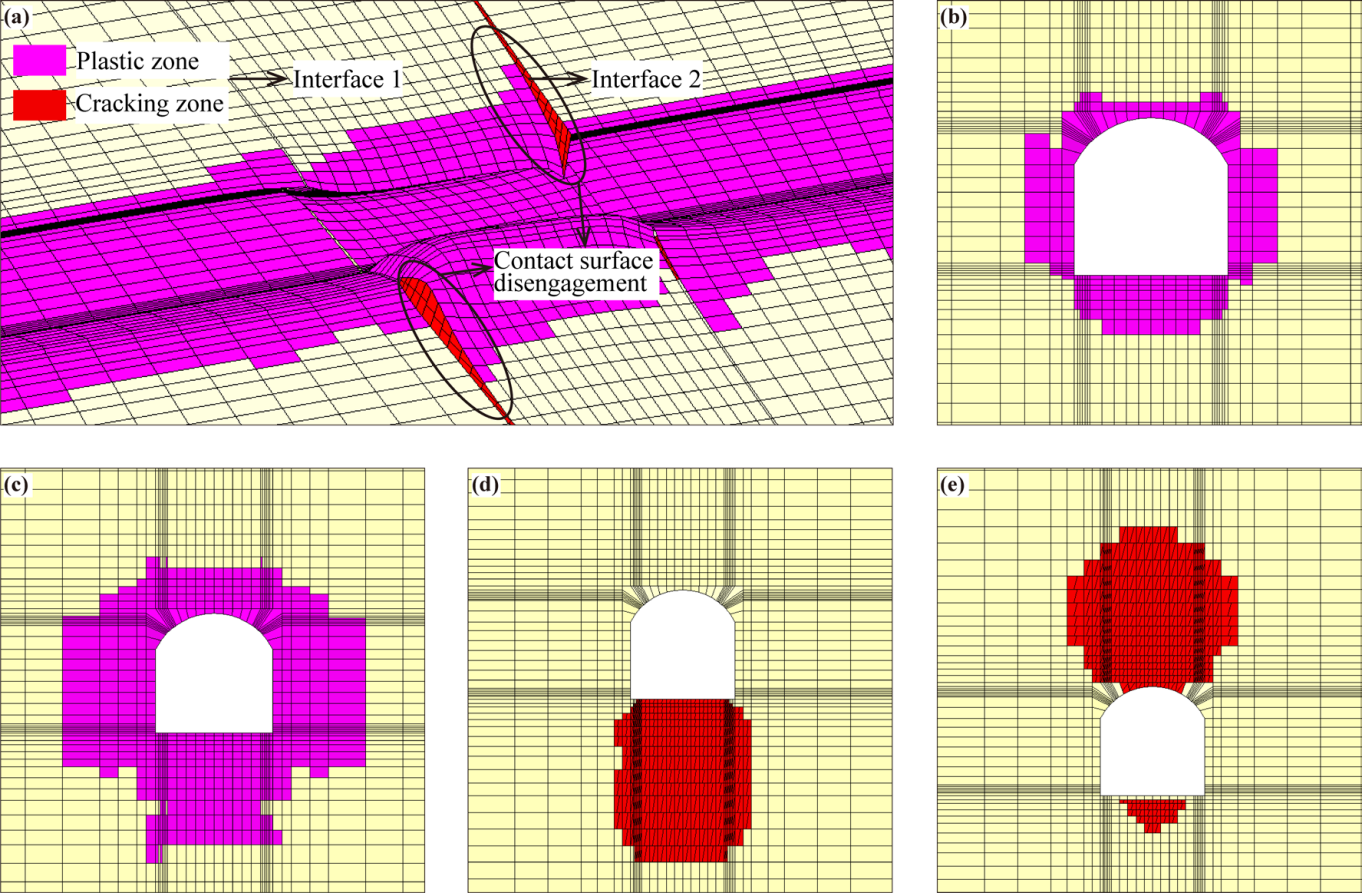
Distribution of the failure zone of the surrounding rock under the condition without support. (**a**) Longitudinal section of the tunnel, (**b**) cross-section of the tunnel (non-fault zone), (**c**) cross-section of the tunnel (fault zone), (**d**) contact surface 1, and (e) contact surface 2.

After excavation, the distributions of the failure zone of the surrounding rock in the tunnel without support are shown in Fig. 13. It can be seen that under the influence of excavation load, the surrounding rock in a certain range around the cave undergoes plastic flow and produces plastic deformation, especially, the range of the plastic zone in the fault zone increases significantly (see Fig. 13[c]). The contact surface is the high-risk area, disengagement damage occurs in some areas (see Fig. 13[a]), in which the disengagement area of contact surface 1 is located at the bottom of the tunnel, while the disengagement area of contact surface 2 is mainly concentrated at the top of the tunnel, which is related to the spatial relationship between the contact surface and the excavation surface of the tunnel.

**Fig. 14:**
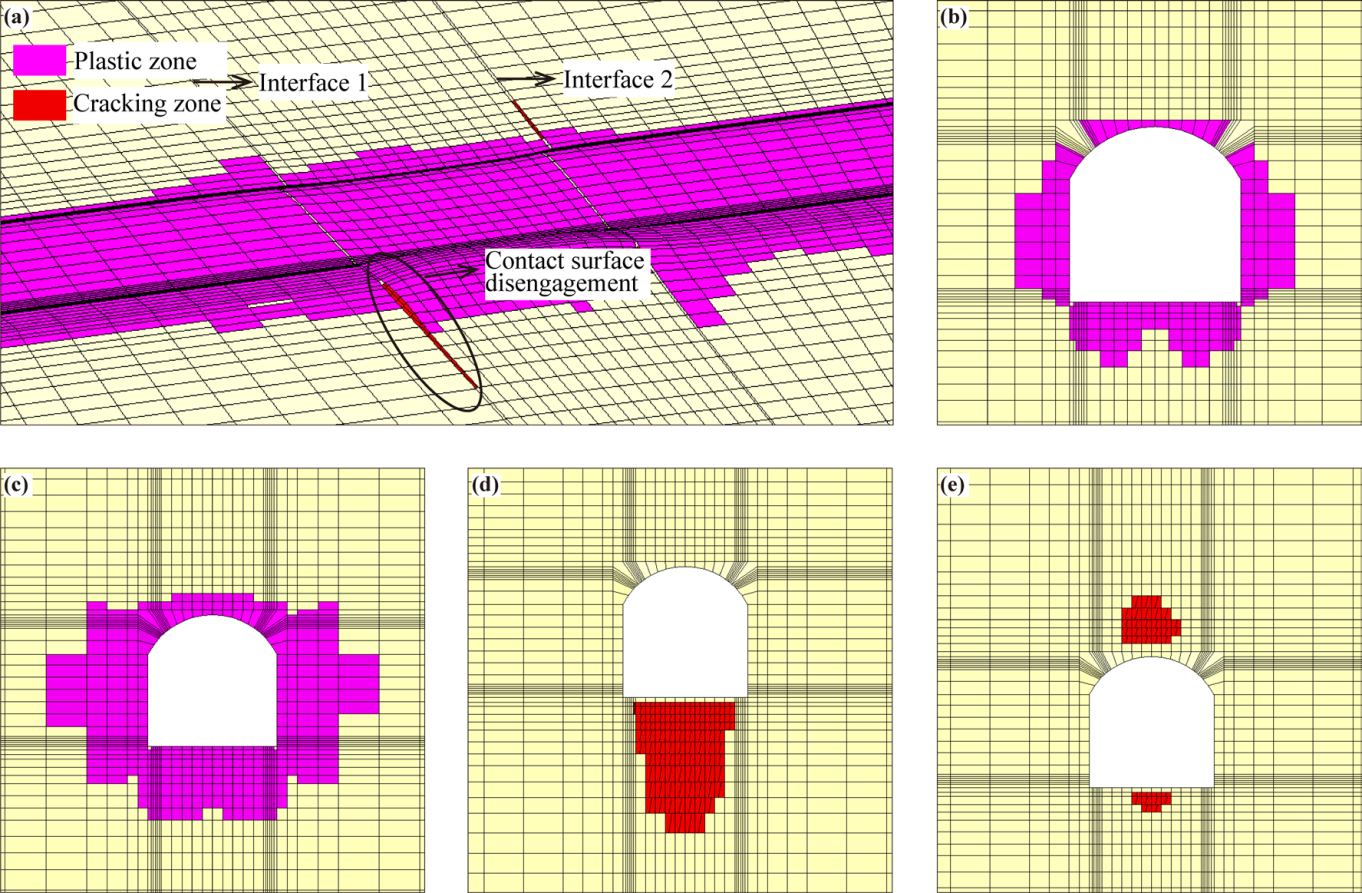
Distribution of the failure zone of the surrounding rock under the condition with support. (**a**) Longitudinal section of the tunnel, (**b**) cross-section of the tunnel (non-fault zone), (**c**) cross-section of the tunnel (fault zone), (**d**) contact surface 1, and (**e**) contact surface 2.

After adopting supporting measures, the distributions of the failure zone of the surrounding rock are shown in Fig. 14, which shows that the distribution law of the failure zone of the surrounding rock is basically the same as that of the condition without support, but the scope of the failure zone is significantly reduced, and the disengagement area of the contact surface is also significantly reduced (see Fig. 14[d]-[e]). The volume of the failure zone of surrounding rock under the condition without support reaches 8473.4 m3, and the volume of the failure zone of surrounding rock is reduced to 6434.3 m3 after taking supporting measures, indicating that the supporting measures significantly limit the rock deformation, which greatly improves the stability of the surrounding rock.

### Analyses of the distribution of the EDC of the surrounding rock in the tunnel

**Fig. 15:**
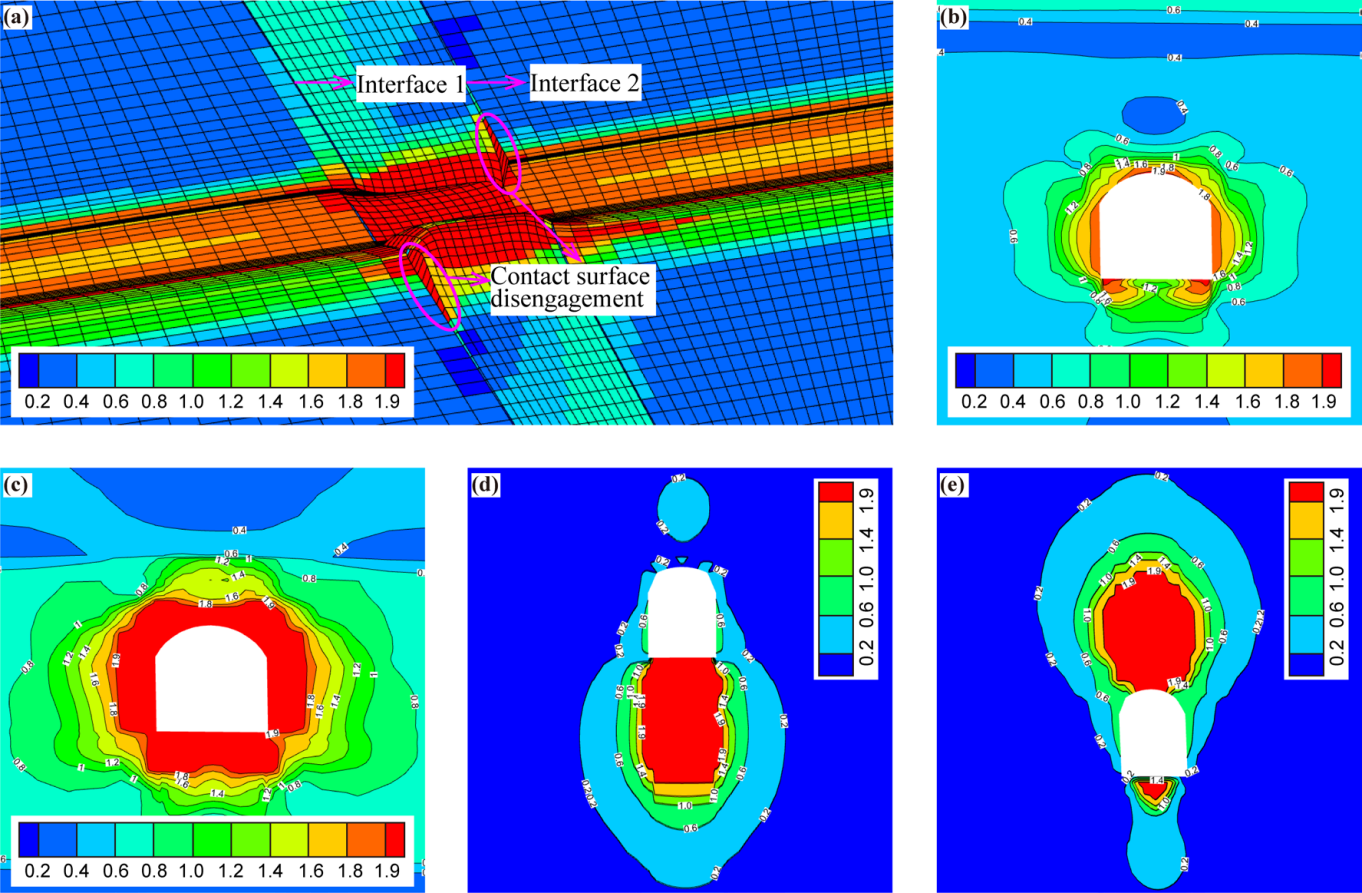
Distribution of the EDC of the surrounding rock under the condition without support. (**a**) Longitudinal section of the tunnel, (**b**) cross-section of the tunnel (non-fault zone), (**c**) cross-section of the tunnel (fault zone), (**d**) contact surface 1, and (**e**) contact surface 2.

The distributions of the EDC of the surrounding rock under the condition without support are plotted in Fig. 15. Figure 15(a)-(c) shows that the values of EDC decrease gradually from the excavation face to the interior rock mass, and the safety degree of the rock element increases gradually. The range of *EDC* = 1.0 fits perfectly with the range of the plastic zone in Fig. 13(a)-(c), and according to whether the value of EDC is greater than 1.0 or not, the surrounding rock can be classified into elastic zone and plastic zone. Figure 15(d)-(e) shows that the contact surface is the weak area, so the EDC value is larger, and the EDC value reaches 2.0 in some areas, which indicates that the contact surface has been disengaged, and the range of *EDC* = 2.0 fits perfectly with the disengagement area of the contact surface in Fig. 13(d)-(e). Figure 15(a) also shows that in the fault zone, the danger degree is obviously higher, and the corresponding EDC value increases significantly compared to that in the non-fault area.

**Fig. 16:**
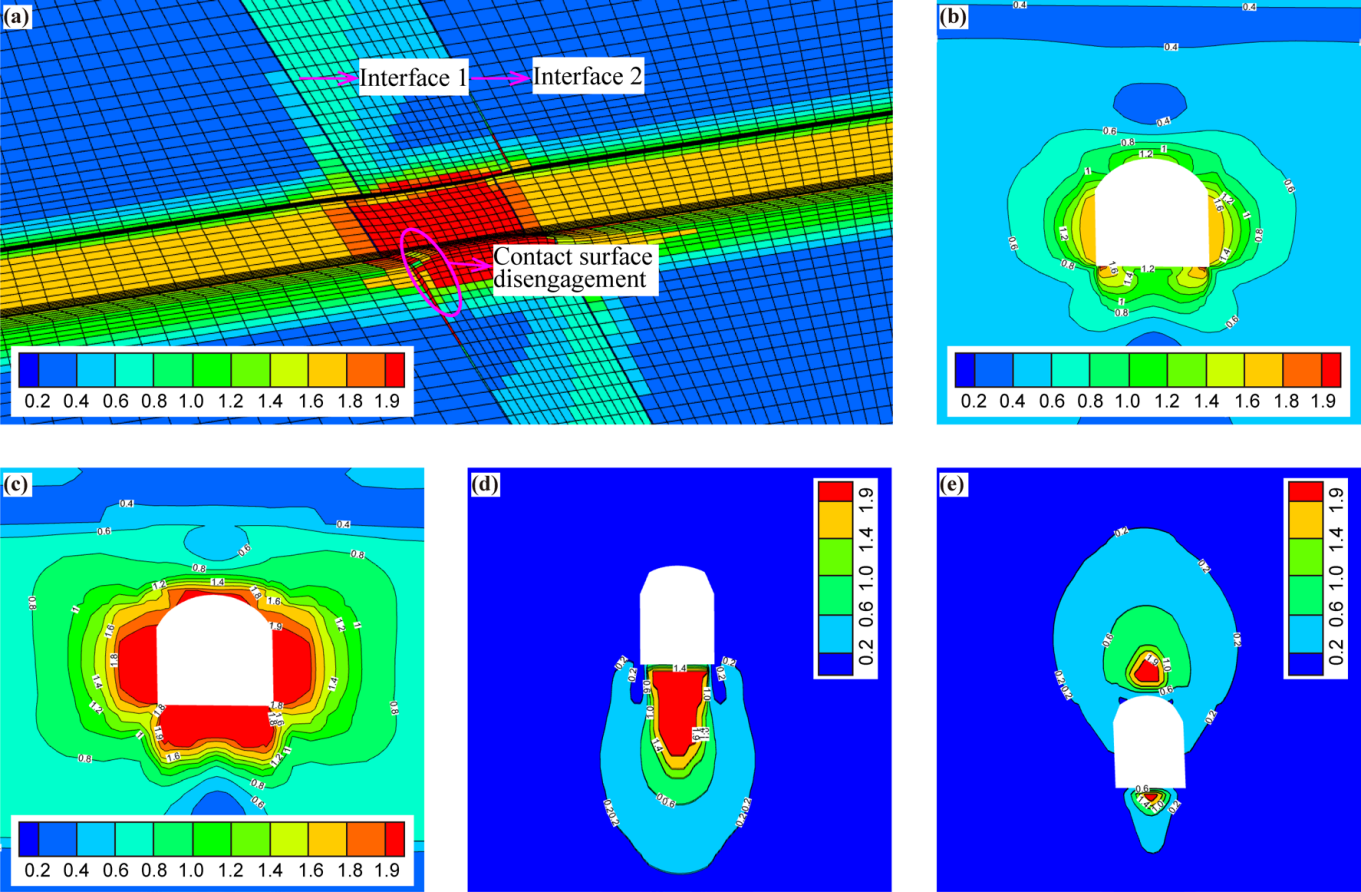
Distribution of the EDC of the surrounding rock under the condition with support. (**a**) Longitudinal section of the tunnel, (**b**) cross-section of the tunnel (non-fault zone), (**c**) cross-section of the tunnel (fault zone), (**d**) contact surface 1, and (**e**) contact surface 2.

After adopting supporting measures, the distributions of EDC of the surrounding rock are plotted in Fig. 16. It can be seen that the range of *EDC* ≥ 1.0 decreases significantly, which indicates that the dangerous degree of the surrounding rock decreases significantly (see Fig. 16[a]-[c]). For the contact surface, the range of *EDC* = 2.0 also decreases significantly, especially the contact surface 2 (see Fig. 16[d]-[e]).

Under the condition without support, *Total_EDC* = 1.81 × 10^5^, after adopting supporting measures, *Total_EDC* = 1.71 × 10^5^. The above results show that it is reasonable to use EDC to quantitatively evaluate the stability of the surrounding rock, and compared with the traditional plastic zone index, EDC can quantitatively evaluate the danger degree of the rock element in the elastic and plastic zones at the same time, and show the element’s dangerous coefficients in the corresponding areas, which is more advantageous than the plastic zone index.

### Application method of EDC in actual engineering projects

In actual engineering projects, for sections with EDC values below 1, they can be considered safe and do not require reinforcement. For sections with EDC values between 1.0 and 2.0, the bearing capacity of the surrounding rock has already decreased, and reinforcement is necessary. For contact surfaces, when EDC reaches 2.0, separation failure will occur, thus requiring special reinforcement measures.

## Conclusions

On the basis of the classical theoretical framework of elastic-plastic mechanics, an elastic-plastic damage constitutive model for rock mass based on the Z-P yield criterion and Drucker-Prager plastic potential function was established by considering the damage of deviatoric stress after yielding. Its correctness was verified by comparing its predicted results with the compression test data for the rock cylinder specimen.

The failure modes of the structural surface were divided into normal disengagement damage and shear damage, and a peak shear strength model of the structural surface was established through rigorous mechanical derivation. On this basis, the elastic-brittle model was adopted to describe the normal tensile behavior of the structural surface, and the tangential nonlinear constitutive relationship of the structural surface was established by considering the post-peak softening behavior and the shear expansion effect of the structural surface. Its rationality was verified by comparing its predicted results with the direct shear test data in the existing literature. By combining it with the thin-layer contact element model, a numerical simulation method for the structural surface was formed.

Based on the existing yielding approaching index, a novel index called the element dangerous coefficient (EDC) was proposed. The proposed method was applied to a cross-fault hydraulic tunnel, and some conclusions were as follows. (1) After excavation, the surrounding rock in a certain range near the excavation surface enters the plastic state, and the element’s EDC value decreases gradually from the excavation surface to the interior rock mass, which indicates that the danger degree of the rock mass decreases gradually. (2) The rock-fault contact surface is the weak area, and the disengagement damage of the contact surface near the excavation surface occurs, where the EDC value reaches 2.0. (3) Compared with the traditional plastic zone index, the EDC can quantitatively evaluate the danger degree of the rock element in the elastic and plastic zones at the same time, and show its dangerous coefficient, which is more advantageous than the plastic zone index.

## Data Availability

The data that support the findings of this study are available from the corresponding author upon reasonable request.

## References

[1] Barton, N. & Choubey, V. The shear strength of rock joints in theory and practice[J]. *Rock. Mech.* **10** (1), 1–54 (1977).

[2] Grasselli, G. *Manuel Rocha Medal Recipient Shear Strength of Rock Joints Based on Quantified Surface Description[J]*39295–314 (Rock Mechanics and Rock Engineering, 2006). 4.

[3] Jaeger, J. C. Friction of rocks and stability of rocks slopes[J]. *Geotechnique* **21** (2), 97–134 (1971).

[4] Plesha, M. E. Constitutive models for rock discontinuities with dilatancy and surface degradation[J]. *Int. J. Numer. Anal. Methods Geomech.* **11** (4), 345–362 (1987).

[5] Leea, H. S. et al. *Influence of Asperity Degradation on the Mechanical Behavior of Rough Rock Joints Under Cyclic Shear loading[J]*38967–980 (International Journal of Rock Mechanics and Mining Sciences, 2001).

[6] Homand, F., Belem, T. & Souley, M. Friction and degradation of rock joint surfaces under shear loads[J]. *Int. J. Numer. Anal. Meth. Geomech.* **25** (10), 973–999 (2001).

[7] Souley, M. & Homand, F. *AMADEI B. An Extension To the Saeb and Amadei Constitutive Model for Rock Joints To Include Cyclic Loading paths[J]*32101–109 (International Journal of Rock Mechanics & Mining Sciences & Geomechanics Abstracts, 1995). 2.

[8] Saeb, S. & Amadei, B. *Modelling Rock Joints Under Shear and Normal loading[J]*29267–278 (International Journal of Rock Mechanics & Mining Sciences & Geomechanics Abstracts, 1992). 3.

[9] Yin, X. J., Wang, G. L. & Zhang, C. H. Study of constitutive model for rock interfaces and joints under Cyclic shear loading[J]. *Eng. Mech.* **22** (6), 97–103 (2005).

[10] Desai, C. S. & Fishman, K. L. *Plasticity-based Constitutive Model with Associated Testing for joints[J]*2815–26 (International Journal of Rock Mechanics & Mining Sciences & Geomechanics Abstracts, 1990). 1.

[11] Wang, J. G., Ichikawa, Y. & Leung, C. F. *A Constitutive Model for Rock Interfaces and joints[J]*4041–53 (International Journal of Rock Mechanics & Mining Sciences & Geomechanics Abstracts, 2003). 1.

[12] Xu, L. et al. Nonlinear elasto-plastic softening constitutive model for rock interfaces and joints[J]. *Chin. J. Appl. Mech.* **25** (3), 462–465 (2008).

[13] Deng, J. *Research on Seismic Response and Shock Absorption Measure for Complex deep-buried Hydraulic tunnel[D]* (Wuhan University, 2017).

[19] Xu, C. H., Ren, Q. W. & Li, R. Advances in researching the stability analysis methods of the surrounding rock mass in underground engineering[J]. *Metal Mine*, (2): 34–37. (2003).

[15] Xie, X. H., Su, B. Y. & Zhan, M. L. Study of failure criterion for brittle rocks based on strains[J]. *Chin. J. Rock Mechan. Eng.* **23** (7), 1087–1090 (2004).

[16] Zhang, Q-Y. et al. Large-scale geo-mechanical model tests for the stability assessment of deep underground complex under true-triaxial stress[J]. *Tunn. Undergr. Space Technol.* **83**, 577–591 (2019).

[17] Zhu, D. R. Criterion for rock project failure[J]. *J. China Coal Soc.* **19** (1), 15–20 (1994).

[18] Sari, Y. D. et al. Numerical analysis of a tunnel support design in conjunction with empirical Methods[J]. *J. Geotech. GeoEnviron. Eng.* **8** (1), 74–81 (2008).

[19] Bhasin, R., Magnussen, A. W. & Grimstad, E. The effect of tunnel size on stability problems in rock masses[J]. *Tunn. Undergr. Space Technol.* **21**, 3–4 (2006).

[20] Zheng, Y. R. et al. Exploration of stability analysis methods for surrounding rocks of soil tunnel[J]. *Chin. J. Rock Mechan. Eng.* **27** (10), 1968–1980 (2008).

[21] Li, S. C., Li, S. C. & Xu, B. S. Minimum safety factor method for stability analysis of surrounding Rockmass of tunnel[J]. *Rock. Soil. Mech.* **28** (3), 549–554 (2007).

[22] Cheng, X-S., Dowding, C. H. & Tian, R-R. New methods of safety evaluation for rock/soil mass surrounding tunnel under earthquake[J]. *J. Cent. South. Univ.* **21** (7), 2935–2943 (2014).

[23] Jang, Q., Feng, X. T. & Xiang, T. B. Discussion on method for calculating general safety factor of underground caverns based on strength reduction theory[J]. *Rock. Soil. Mech.* **30** (8), 2483–2488 (2009).

[24] Yuan, M. et al. Z-P yield criterion based analysis of the element safety factor for the stability of surrounding rock[J]. *Mod. Tunn. Technol.* **52** (5), 48–53 (2015).

[25] Xie, H. P., Peng, R. D. & Ju, Y. Energy dissipation of rock deformation and fracture[J]. *Chin. J. Rock Mechan. Eng.* **23** (21), 3565–3570 (2004).

[26] Cai, M. F., Kong, G. Y. & Jia, L. H. Criterion of energy carastrophe for rock project system failure in underground engineering[J]. *J. Univ. Sci. Technol. Beijing*. **19** (4), 325–328 (1997).

[27] Xu, C. H. & Ren, Q. W. Criterion of entropy catastrophe of stability of surrounding rock[J]. *Rock. Soil. Mech.* **25** (3), 437–440 (2004).

[28] Ma, S. & Xiao, M. Judgment method for stability of underground cavern based on catastrophe theory and monitoring displacement[J]. *Chin. J. Rock Mechan. Eng.* **29** (S2), 3812–3819 (2010).

[29] Fu, C. H. & Cheng, S. H. Study on instability criteria of surrounding rock of underground engineering cavern based on catastrophe theory[J]. *Rock. Soil. Mech.* **29** (1), 167–172 (2008).

[30] Desai, C. S. et al. Thin-layer element for interfaces and joints[J]. *Int. J. Numer. Anal. Meth. Geomech.* **8**, 19–43 (1984).

[31] Frantziskonis, G. & Desai, C. S. Constitutive model with strain softening[J]. *Int. J. Solids Struct.* **23** (6), 733–768 (1987).

[32] Zienkiewicz, O. C. & Panda, G. N. Some useful forms of isotropic yield surfaces for soil and rock mechanics[Z]//GODEHUS G. Proceedings of Finite Elements in Geomechanics. New York; John Wiley and Sons Press. : 179–190 (1977).

[33] Wang, J. X. & Jiang, A. N. An elastoplastic damage constitutive model of rock and its application to tunnel engineering[J]. *Rock. Soil. Mech.* **36** (4), 1147–1158 (2015).

[34] Wang, X. W. et al. Nonlinear finite element simulation method for contact face of rock-anchored beam and surrounding rock in underground house[J]. *Adv. Eng. Sci.* **49** (04), 70–77 (2017).

[35] Pande, G. N. & Sharma, K. G. *On joint/interface Elements and Associated Problems of Numerical ill-conditioning[J]*3293–300 (International Journal of Rock Mechanics & Mining ences & Geomechanics Abstracts, 1979). 3.

[36] Samadhiya, N. K., Viladkar, M. N. & Al-Obaydi, M. A. Three-Dimensional joint interface element for rough undulating major discontinuities in rock Masses[J]. *Int. J. Geomech. ASCE*. **8** (6), 328–331 (2008).

[37] Li, H. B. et al. Study of deformability behaviour and failure mechanism by simulating rock joints sample under different loading conditions[J]. *Rock. Soil. Mech.* **29** (7), 1741–1752 (2008).

[38] Li, X. F. et al. Numerical simulation of mechanical characteristics of jointed rock in direct shear test[J]. *Rock. Soil. Mech.* **37** (2), 583–591 (2016).

[39] Zhou, H. *Numerical Simulation for Seismic Joint Response of Surrounding Rock and Supporting Structure of Underground caverns[D]* (Wuhan University, 2017).

[40] Luan, M. T. & Wu, Y. J. A nonlinear elasto-perfectly plastic model of interface element for soil-structure interaction and its applications[J]. *Rock. Soil. Mech.* **25** (4), 507–513 (2004).

[41] Grassellia, G. & Eggerb, P. *Constitutive Law for the Shear Strength of Rock Joints Based on three-dimensional Surface parameters[J]*4025–40 (International Journal of Rock Mechanics & Mining Sciences & Geomechanics Abstracts, 2003).

[42] Lee, S. W. *Stability around Underground Openings in Rock with dilative, non-persistent and multi-scale Wavy Joints Using a Discrete Element method[D]* (The University of Illinois, 2003).

[43] Zhou, H. et al. Analysis of rock mass stability in tunnel and underground engineering based on yield approach index[J]. *Chin. J. Rock Mechan. Eng.* **24** (17), 3083–3087 (2005).

[44] Gao, L. Y. et al. Analysis and application of yielding approach based on material strength criteria[J]. *J. Chongqing Univ.* **39** (5), 73–81 (2016).

[45] Liu, H. & Xiao, M. Stability assessment of surrounding rock of underground cavern complexes based on energy-dissipation model; proceedings of the Asia-Pacific Power and Energy Engineering Conference, APPEEC 2010, Chengdu, China, F, 2010 [C]. IEEE Computer Society.

[46] Xiao, M. *Study on Numerical Analysis Method of Stability and Supporting for Underground Caverns [D]* (Wuhan University, 2002).

[47] Kong, C. et al. A numerical model for fully grouted rock bolts considering the nonlinearities of the bolt and the bolt-grout interface[J]. *Comput. Geotech.*, 160, 105522. (2023).

[48] Kong, C. et al. Numerical simulation of fully grouted rock bolts with or without faceplates based on the tri-linear bond-slip model[J]. *Constr. Build. Mater.*, 367, 130288. (2023).

[49] Kong, C., Xiao, M. & Yuan, Q. Implicit nonlinear FEM for steel sets in tunnels[J]. *Sci. China Technological Sci.* **66**, 771–783 (2023).

[50] Cen, W. J. et al. Numerical simulation of seismic damage and cracking of concrete slabs of high concrete face rockfill dams[J]. *Water Sci. Eng.* **9** (3), 205–211 (2016).

[51] Bandis, S. & Lumsden, A. Experimental studies of scale effects on the shear behaviour of rock joints. *Int. J. Rock. Mech. Sci. Geomech. Abstracts*. **18**(1), 1-21 (1981).

